# Matrix Metalloproteinase‑9 (MMP-9) Activatable Gold Nanoparticles for *In Situ* Zymography and Diagnostics of Neurofibromatosis Type 2 (NF2) Tumors

**DOI:** 10.1021/acsanm.5c04657

**Published:** 2026-01-23

**Authors:** Shimayali Kaushal, Han T. N. Nguyen, Melanie Fisher, Hsuan-Chih Kuo, Zachary D. Schultz, Yin Ren

**Affiliations:** † Department of Otolaryngology−Head and Neck Surgery, Division of Otology, Neurotology and Cranial Base Surgery, 12306 The Ohio State University Wexner Medical Center, Columbus, Ohio 43210, United States; ‡ Department of Chemistry and Biochemistry, The Ohio State University, Columbus, Ohio 43210, United States

**Keywords:** vestibular schwannoma, matrix metalloproteinase-9, gold nanoparticles, *in situ* zymography, tumor diagnostics

## Abstract

Neurofibromatosis type 2-related schwannomatosis (NF2-SWN) is a devastating genetic tumor-disposition syndrome characterized by multiple nervous system neoplasms. A hallmark of NF2-SWN is bilateral vestibular schwannomas (VSs) that cause hearing loss, vertigo, and life-threatening brainstem compression. Current imaging methods detect NF2-associated VS often late in their development and could lead to delayed therapeutic interventions. Matrix metalloproteinase-9 (MMP-9) is a key protease involved in extracellular matrix remodeling and tumor progression in NF2-associated VS, making it an attractive molecular target for activity-based sensing. Here, we develop MMP-9 activatable gold nanoparticles (AuNPs) incorporating a protease-cleavable peptide to enable sensitive reporting of protease activity in VS tissue. Through systematic tuning of polyethylene glycol (PEG) linker length and peptide valency, we establish an AuNP configuration that exhibits both efficient enzymatic cleavage and selectivity for MMP-9 over other VS-associated proteases. In schwannoma tissue, nanoparticles distinguish tumor from healthy nerve with >95% accuracy and detect 2 mm tumors—corresponding, based on published VS growth rates, to detection approximately 23 months earlier than conventional MRI—enabling substantially earlier intervention. Longitudinal measurement of nanoparticle activation further resolves changes in MMP-9 activity within the tumor in response to therapeutic intervention, illustrating the platform’s capacity in monitoring treatment response. Together, these findings establish MMP-9 activatable AuNP as a sensitive, spatially resolved diagnostic tool for NF2-SWN that complements imaging by directly quantifying protease activity in tumors that are inaccessible to biopsy.

## Graphical abstract

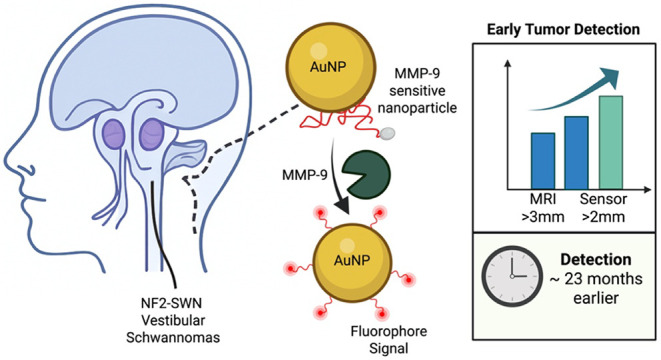

## Introduction

NF2-related schwannomatosis (NF2-SWN, also known as neurofibromatosis type 2) is a devastating genetic disorder characterized by the development of multiple tumors throughout the nervous system, which significantly and negatively impact a patient’s quality of life. The burden of NF2-associated VS is profound, causing speech and swallowing difficulties, hydrocephalus, and cognitive decline.^1^ Tumor compression of the spinal cord can result in paralysis or loss of motor function.^2^ Bilateral vestibular schwannomas (VSs), the hallmark tumor of NF2-SWN, can lead to profound sensorineural hearing loss, vertigo, and brainstem compression. Given that VS and most other NF2-associated tumors are inaccessible for biopsies, there are currently few biomarkers to predict tumor progression or response to treatment, making management of NF2-associated VS particularly challenging.^3^ Immunohistochemical or molecular analysis of tumor specimens, although informative, may not accurately reflect tumor biology due to high intratumoral heterogeneities and is not applicable to nonsurgical patients. Current clinical management of VS is primarily governed by magnetic resonance imaging (MRI) or progression of hearing loss,^4,5^ which suffers from delayed diagnosis, missed treatment opportunities, and potentially suboptimal outcomes. Therefore, there is a critical need to develop new diagnostic tools that can detect VS at an earlier stage, predict tumor growth before changes are evident on imaging, and determine drivers of tumor progression.

The VS tumor microenvironment (TME) plays a critical role in promoting tumor growth, enhancing migratory potential, and driving therapeutic resistance,^6–8^ highlighting the need for methods that can assess these dynamic changes in real time. The VS microenvironment consists of extracellular matrix (ECM), cytokines, and proteases, all of which influence tumor progression.^9–12^ Dysregulated protease activity contributes to tumor growth, invasion, angiogenesis, and immune evasion, making them ideal biomarkers of tumor behavior.^13–15^ The use of engineered peptides enables activity-based detection by incorporating protease-cleavable linkers that give a signal only in the presence of tumor-associated protease activity, thereby offering higher specificity than conventional approaches.^16–18^ Developing better methods to screen peptide substrates and map tumor protease activity can improve the specificity and clinical use. Recent progress in MMP-responsive nanosensors and activity-based detection platforms underscores growing interest in protease-driven diagnostic strategies.^19–21^ Conventional analytical methods—ELISA, gelatin zymography, and fluorescent probes—are limited by labor-intensive protocols, an inability to distinguish active from inactive enzymes, incompatibility with intact tissues, and a lack of quantitative or spatial information.^22–25^ Peptide-based molecular beacons and activatable imaging probes enable MMP activity detection^26,27^ but often suffer from limited tissue penetration, rapid clearance, or insufficient amplification. Nanoparticle-assisted activatable sensors are promising by enhancing stability and sensitivity,^28,29^ yet none have been applied to tumors such as vestibular schwannoma. Gold nanoparticles have been widely used in enzymatic sensing because of their tunable optical properties, ease of surface modification, and high signal amplification capability.^30–32^ Among proteases, MMP-9 is crucial in angiogenesis, ECM remodeling, and metastasis.^33^ In VS, elevated MMP-9 levels correlate with aggressive features like rapid growth and development of peritumoral adhesions.^34^ Detecting and monitoring MMP-9 activity in real time and *in situ* within schwannoma tissue can provide valuable insights into tumor biology without the need for invasive biopsies.^16,17,35^ However, its clinical utility as a plasma biomarker is limited due to its endogenous nature, high background interference, and variable sensitivity. In contrast, MMP-9 as a tissue biomarker offers a more spatially specific approach for identifying active tumor regions.^36^ To address this, we employed a synthetic biomarker strategy to generate a detectable fluorescent signal at the tumor site, detecting protease activity in real time with greater sensitivity and specificity.^37^

To address the limited precision of current diagnostics for NF2-associated VS, we developed peptide-functionalized AuNPs to amplify and spatially localize tumor-specific MMP-9 activity in VS patients. This approach enhances the signal-to-noise ratio by amplifying endogenous protease activity, enabling tumor detection before morphological changes are detected on MRI, and improving therapeutic response monitoring. We further validate the platform using two schwannoma mouse models, demonstrating its ability to noninvasively track MMP-9 activity, tumor growth, and response to MMP-9 inhibition. By enabling noninvasive and quantitative measurement of tumor-associated protease activity, this system provides key mechanistic insights and supports personalized decision-making for NF2-SWN patients.

## Results and Discussion

In this study, we developed a protease activity-based nanoparticle platform to detect and localize matrix metalloproteinase-9 activity. We applied the system to VS, a rare intracranial tumor located in the skull base and a hallmark of Neurofibromatosis Type 2, a debilitating genetic tumor predisposition syndrome with no cure. VS is inaccessible for biopsy due to its intracranial location, and standard diagnostic tools such as MRI lack resolution for detecting subcentimeter tumors or monitoring tumor growth and treatment response. There is an urgent unmet need for agents that can detect molecular drivers of tumor progression and predict which tumors will grow, as early identification of growing VS can improve patient counseling, reduce unnecessary imaging, and potentially improve clinical outcomes. We screened a library of protease substrates and identified a peptide preferentially cleaved by the VS-secreted MMP-9. Next, we applied our nanoparticle as an *in situ* zymography sensor to schwannoma specimens from a mouse model and VS patients undergoing surgical resection, where it quantified MMP-9 activity with high spatiotemporal resolution and distinguished tumor from healthy nerve. We deployed the nanoparticles *in vivo* and demonstrated that MMP-9 activation enables tracking of subcentimeter tumor growth, surpassing limitations of current imaging modalities. This heightened sensitivity not only facilitates earlier tumor detection but also empowers monitoring of the therapeutic response to protease inhibition. Further, we demonstrated this nanosensor can localize to tumor margins and characterize key hallmarks associated with VS progression, including angiogenesis and infiltration of macrophages and activated fibroblasts. Lastly, we showed nanoparticles enable real-time monitoring of response to protease inhibition, which effectively attenuated NF2-associated VS growth *in vivo*.

### Identification of MMP-9-Cleavable Peptides

We aim to develop a nanoparticle sensor to spatiotemporally profile the MMP-9 activity during VS progression. First, we set out to identify a peptide substrate that is efficiently and specifically cleaved by MMP-9. The EDC/NHS coupling chemistry used for peptide conjugation and the design of the peptide-AuNP are shown in Figure 1A,B, where the fluorophore-quencher-peptide motif is coupled to the nanoparticle surface to enable MMP-9-dependent fluorescence activation.

**Figure 1:**
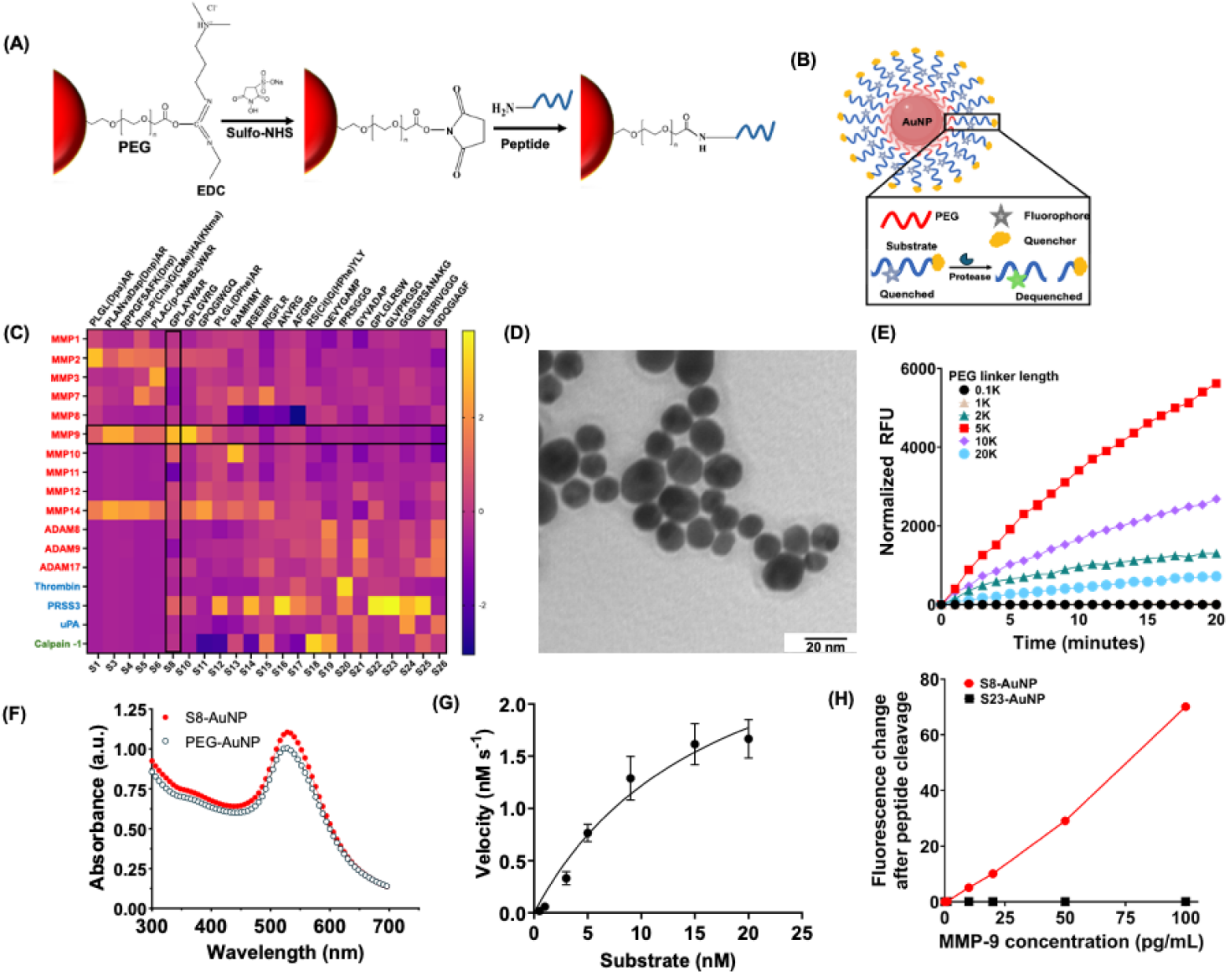
Characterization of peptide-conjugated AuNPs. (A) Scheme for peptide conjugation to AuNPs via EDC/NHS coupling. (B) Schematic depicting the cleavage of peptide-AuNPs by proteases. (C) Heatmap of peptide cleavage activity displayed as z-scores. Each box corresponds to the normalized activity value. Highlighted black-boxed regions mark the intersection of the S8 peptide row with the MMP-9 column, indicating strong cleavage by MMP-9. (D) Transmission electron micrograph (TEM) of AuNPs. Scale bar = 20 nm. (E) Fluorescence intensity of peptide-AuNPs with various PEG lengths, demonstrating the highest efficiency with a 5 kDa PEG linker (red). (F) Absorbance spectra of PEGylated AuNPs (open circle) and S8-AuNPs (red circle). (G) Michaelis–Menten enzymatic cleavage kinetics of the S8 peptide. (H) Fluorescence-based cleavage of peptide-AuNPs using MMP-9. Relative fluorescence intensities of S8-AuNP (red) and S23-AuNP (black) vs MMP-9 (0–100 pg/mL).

We designed a custom library consisting of 26 peptides, including sequences that encompass cleavage sites of 17 common tumor-associated proteases.^38–42^ Each peptide is flanked by an N-terminal FAM (6-carboxyfluorescein) fluorophore and a C-terminal quencher (CPQ2) based on their consistent spectral overlap for Förster resonance energy transfer (FRET) (Table S1). Protease-mediated cleavage disrupted the peptide bond at the cleavage site and led to increased fluorescence over time, enabling selection of peptides with the highest cleavage efficiency and specificity for each protease (Figure 1C). Among the peptide candidates, S10 generated the highest fluorescence signal by MMP-9; however, it also showed substantial cleavage by MMP-2, MMP-10, and PRSS3, indicating poor specificity. In contrast, S8 (GPLAYWAR) showed strong MMP-9 cleavage efficiency with low activity toward MMP-2, MMP-10, and PRSS3. It has a Z-score of 0.53, a measure of cleavage efficiency based on fluorescence signal strength, with MMP-9 accounting for over half of the signal. S8 displayed 6.5-fold, 2.6-fold, and 2.9-fold greater selectivity for MMP-9 than MMP-2, MMP-10, and PRSS3, respectively, with minimal activation by related proteases on interference analysis. Future work will evaluate additional interferents, such as serum and extracellular matrix proteins, to validate assay specificity under more complex conditions.

### Optimization of MMP-9 Sensing Nanoparticles for Enhanced Performance

Following the identification of an MMP-9-cleavable FRET peptide, we hypothesized that S8 sensitivity against MMP-9 and specificity over other proteases can be further optimized via multivalent peptide presentation on a gold nanoparticle (AuNP) surface. Here, gold was selected due to its biocompatibility, inertness, and well-defined surface chemistry, which enables stable and reproducible peptide conjugation. Unlike silver, iron oxide, or polymeric nanoparticles, which can be cytotoxic, require complex surface coatings, and lack precise control over surface coatings required for predictable FRET-based designs, gold provides a robust and biocompatible platform for enzymatically activatable sensing.^31^ AuNPs were functionalized using polyethylene glycol (PEG) to improve biocompatibility and colloidal stability via EDC/NHS coupling of the peptide’s amine group to the carboxyl-terminated PEG. We hypothesized that iterative optimization of both the distance from the peptide to the nanoparticle core and peptide valency would tune the specificity between on- and off-target substrate cleavage. Transmission Electron Microscopy (TEM) confirmed that the AuNPs were monodisperse (Figure 1D). AuNPs were tested with different PEG lengths, with 5 kDa PEG demonstrating the highest fluorescence increase (Figure 1E). We systematically varied the peptide valency from 1:5 to 1:15 (AuNP:peptide) and tuned the distance from the peptide to the AuNP core and found a valency of 1:12 (Figure S1A) with 5 kDa PEG resulted in maximum on-target fluorescence activation. Successful peptide conjugation was confirmed via agarose gel electrophoresis, where peptide-AuNP exhibited slower mobility than unconjugated AuNP (Figure S1B). UV–vis spectroscopy demonstrated a red spectral shift (Figure 1F), indicating changes in the surface plasmon resonance of the AuNP due to peptide conjugation. Dynamic light scattering (DLS) confirmed an increase in apparent dynamic size from 20 to 42 nm and a surface zeta potential of −32 mV (Figure S1C). The assay exhibited a time-dependent fluorescence curve Figure S1D) and a linear response within the 0–100 pg/mL range (Figure S1E). Relative standard deviation (RSD) values (*n* = 3) at representative MMP-9 concentrations were within acceptable variability, confirming good assay repeatability (Table S2).

Understanding the proteolytic kinetics of MMP-9 is essential for optimizing the design of activity-based sensors to improve the catalytic efficiency and affinity. We measured S8 concentration dependence on MMP-9 cleavage velocity and fitted the data to the Michaelis–Menten equation, showing a *K*_m_ of 16.92 × 10^–9^ M and a catalytic efficiency (*K*_cat_/*K*_m_) of 1.93 × 10^7^ M^–1^ s^–1^ (Figure 1G). Due to the nonspecificity of several commercial substrates toward MMP-9, their kinetic profiles show poor Michaelis–Menten fitting and unreliable *K*_m_ estimation.

We next compared the limit of detection (LOD) between S8-AuNPs and a commercially available MMP-9 ELISA. AuNPs bearing S23 were used as a control, as S23 has the same length as S8 but is minimally cleaved by MMP-9. Increasing concentrations of MMP-9 were incubated with S8-AuNPs and S23-AuNPs. S8-AuNPs exhibited a detectable fluorescence increase at 10 pg/mL (0.109 pM), indicating the limit of detection (LOD). In contrast, S23-AuNPs showed negligible activation across all MMP-9 concentrations (Figure 1H). The sensitivity of S8-AuNPs is nearly 50% higher than commercial ELISAs, which typically report LOD values in the range of 22–156 pg/mL. Furthermore, S8-AuNPs significantly reduced the labor associated with sample preparation steps, accelerating a 3-h ELISA assay to under 15 min. Further, S8-AuNPs are fundamentally different, as they function as activity-based sensors that measure MMP-9 enzymatic activity, in contrast to ELISA, which quantifies the total amount of protease. The superior sensitivity and streamlined workflow highlight the potential of nanoparticles as a powerful tool for MMP-9 detection for *in vivo* applications. Although our platform exhibited lower LOD than the commercial assay, differences in matrix interference, enzyme incubation time, and assay format can influence this sensitivity. Future optimization and side-by-side testing under identical conditions will be necessary to fully validate the extent of the assay improvement.

### Cellular Uptake and Colocalization with Endosome Markers

We next investigated nanoparticle uptake in an NF2 mouse schwannoma cell line (MD-MSC) that secretes MMP-9.^34^ There was minimal cytotoxicity and >95% viability with S8-AuNPs and S23-AuNPs at up to 500 nM (Figure 2A). A slight increase in cell viability at higher concentrations of S23-AuNPs is likely due to assay variability and differences in nanoparticle dispersion or sedimentation rather than cytotoxicity. Overall, these data indicate that both formulations are well tolerated by schwannoma cells. We quantified the internalization of S8-AuNPs by confocal microscopy, which increased linearly from 1 to 4 h and plateaued thereafter (Figure 2B and Figure S2). S8-AuNPs exhibited efficient MMP-9 cleavage, resulting in quencher release and a marked fluorescence increase, whereas S23-AuNPs showed minimal fluorescence (Figure 2C). To validate that fluorescence was due to MMP-9, cells were treated with Marimastat, a broad-spectrum protease inhibitor, which resulted in a 15-fold decrease in fluorescence (Figure 2D). To study the mechanism of cellular internalization, we examined the intracellular colocalization of early and late endosomal markers with S8-AuNPs. Mander’s coefficient analysis demonstrated a coefficient of 0.81 for Rab4 (a marker of early endosomes) and 0.67 for Rab11 (a marker of late endosomes, Figure 2E), suggestive of excellent overlap. Together, these findings suggest that S8-AuNPs demonstrate excellent biocompatibility and efficient endocytosis, highlighting their potential for protease-activatable sensing in MMP-9-rich environments.

**Figure 2:**
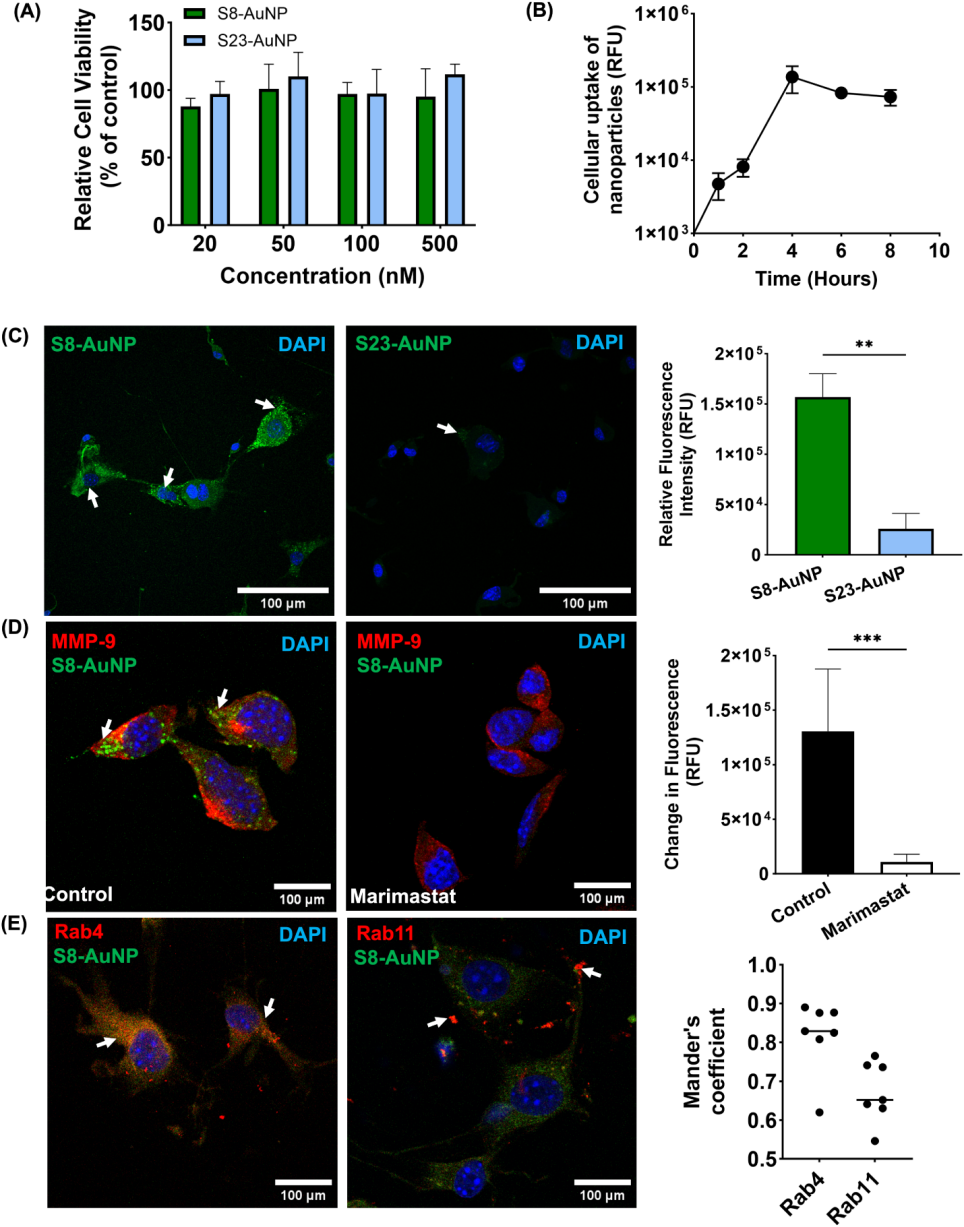
*In vitro* evaluation of peptide-conjugated gold nanoparticles. (A) Cell viability assessed by MTT assay following treatment with varying concentrations of peptide-AuNPs. (B) Cellular uptake of AuNPs, demonstrating maximum uptake at 4 h. (C) Immunocytochemistry and quantification comparing fluorescence of S8-AuNPs and S23-AuNPs (green). **p < 0.01. (D) IF and quantification of S8-AuNPs (green) in the absence (Control; left) or presence of marimastat (right). MMP-9 (red), ***p < 0.001. (E) IF and Mander’s coefficient quantification showing colocalization of S8-AuNPs (green) with endosome markers (Rab4 and Rab11, red). Scale bar: 100 μm. DAPI (blue) for all IF images. Statistical significance was assessed using the Mann–Whitney *U* test.

### *In Situ* Zymography and Tumor Protease Imaging

MMP-9 is produced by schwannoma cells, stromal cells, and infiltrating immune cells^9^ and its expression has been localized to the cystic VS tumor wall, peritumoral vasculature, and tumor margins.^34,43^ To investigate the ability of S8-AuNPs to classify tumor from nontumor tissue, we developed a modified *in situ* zymography approach that overcomes limitations of bulk zymography and ELISA by preserving tissue architecture and enabling detailed, spatially resolved enzyme activity assessment. To investigate the feasibility of capturing MMP-9 activity in fresh tumor specimens obtained from surgical resection, we first synthesized a peptide sequence consisting of a cationic poly-R region complexed with an anionic poly-E region via an MMP-9 cleavable peptide linker (S8Z) and subsequently conjugated it to PEG-AuNPs (Figure 3A and Table S1). Cleavage of S8Z by MMP-9 releases the cationic domain, which binds to negatively charged tissue via electrostatic interactions. While previous studies^44–47^ have shown that solid tumors possess a negatively charged ECM enriched in sulfated glycosaminoglycans and sialylated glycoconjugates, we did not directly measure ECM charge in our schwannoma tissues. The same poly-R/poly-E peptide connected by a non-MMP-9 cleavable D-amino acid sequence was used as a control (dS8Z) (Table S1). Tumors treated with S8Z-AuNP showed a nearly 4-fold increase in fluorescence compared to dS8Z-AuNP, suggesting that MMP-9 remained enzymatically active (Figure 3B,C). The nanoparticle enabled precise spatial localization of MMP-9 activity in VS tissue with minimal background. No fluorescence signal was seen in normal sciatic nerve, and the addition of a small molecule MMP-9 inhibitor (MMP-9-IN-I) significantly reduced S8Z-AuNP binding (Figure 3D,E). Across 6 tumors examined, S8Z-AuNP demonstrated significantly enhanced detection of MMP-9 (area under the curve [AUC] by receiver operating characteristic [ROC] = 1.0; *p* = 0.009; Figure 3F), indicating the strong diagnostic capability of S8Z-AuNP. The ROC curve is based on *in situ* zymography measurements from a small cohort (*n* = 6) of genetically identical mouse schwannoma tissues and paired normal nerves. The perfect AUC value of 1.0 likely reflects the low variability inherent in this controlled data set. Future work will expand the sample size and include tumors of different sizes, as well as more heterogeneous controls, to rigorously evaluate the diagnostic accuracy across diverse biological contexts.

**Figure 3:**
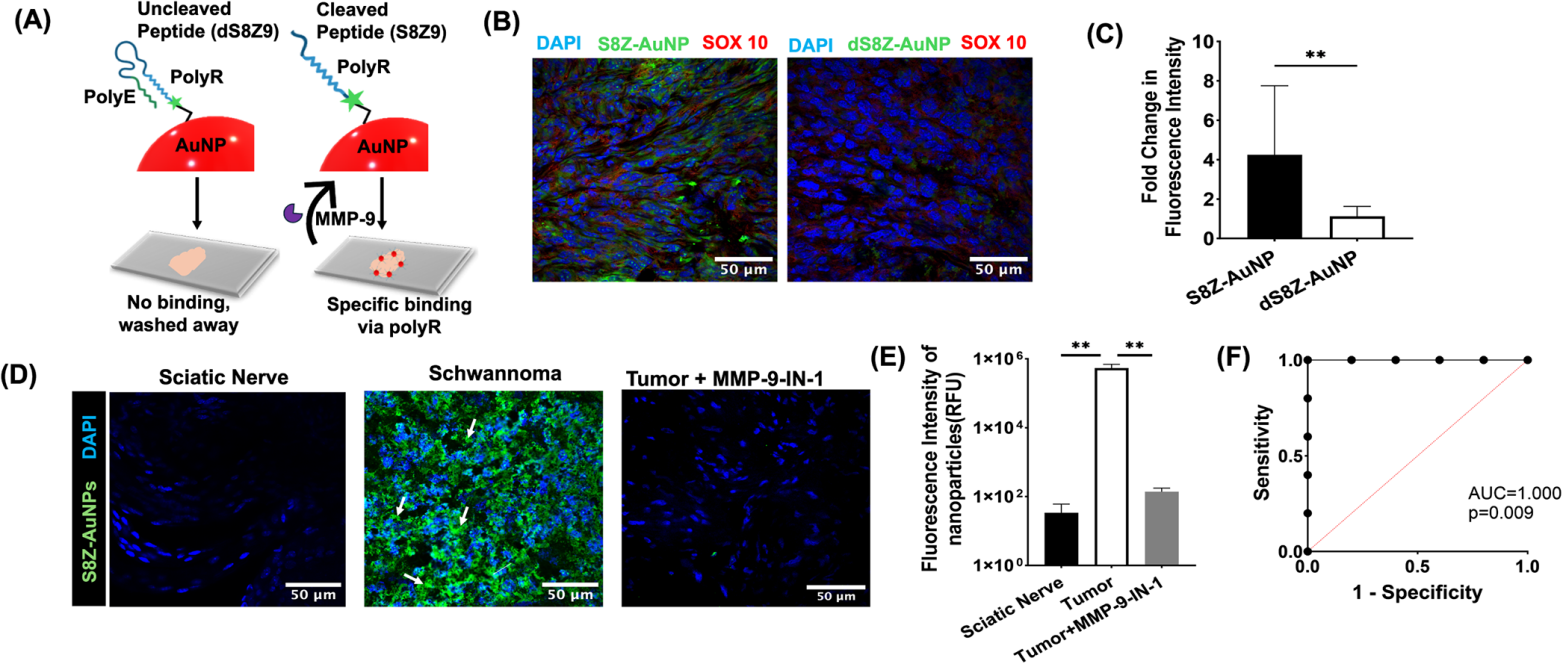
*In situ* localization of tumor MMP-9 activity. (A) Schematic illustrating the interaction-based binding of S8Z-AuNPs to tumor sections. (B) IF showing the fluorescence of S8Z-AuNPs compared to dS8Z-AuNPs; DAPI (blue), SOX10 (red), nanoparticle (green). Scale bar: 50 μm. (C) Quantification of fold-change in fluorescence intensity of S8Z-AuNPs and dS8Z-AuNPs; **p < 0.01. (D,E) Confocal imaging and quantification illustrating the classification of normal nerve from tumor using S8Z-AuNPs (green) and inhibition by MMP-9 IN-1. Scale bar: 50 μm; **p < 0.01. Statistical significance for all comparisons was assessed using the Mann–Whitney *U* test. (F) ROC indicates perfect classification between diseased and healthy states; AUC = 1.0, **p = 0.009.

It has been demonstrated that MMP-9 levels rise significantly as tumors grow, particularly in adherent tumors where invasiveness and ECM remodeling are more prominent.^34^ The ability of MRI to detect millimeter increases in tumor size is limited due to measurement variability and the slow natural history of tumor progression. We next assessed whether S8Z-AuNP-enhanced MMP-9 labeling could serve as a biomarker of VS growth, even prior to radiographic progression becomes evident. In human VS histological sections, fluorescence of S8Z-AuNP increased linearly with tumor volume (Figure 4A). Tumors treated with MMP-9-IN-I showed markedly reduced fluorescence, confirming that the signal is primarily due to MMP-9 activity. S8Z-AuNP’s high signal-to-noise ratio allows it to differentiate tumors with as little as a 0.07 cm^3^ volume difference. The linear correlation between fluorescence intensity and tumor volume suggests that the S8Z-AuNP can detect growth-associated molecular changes with greater sensitivity that could be missed on MRI (Figure 4B).

**Figure 4:**
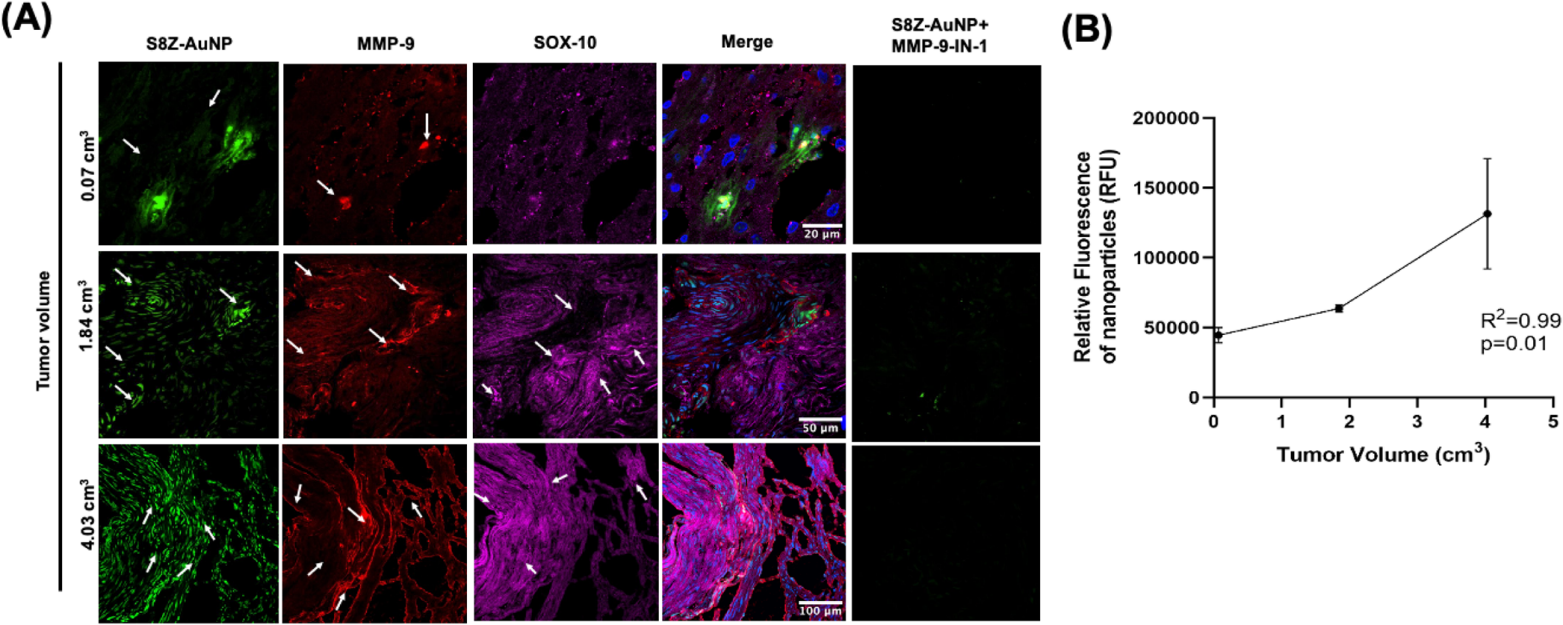
*In situ* mapping of MMP-9 activity in VS tissue. (A) IF showing increased MMP-9 and S8Z-AuNP fluorescence correlated with tumor size. MMP-9-IN-I abrogated S8Z-AuNP fluorescence signal across all tumors; DAPI (blue), nanoparticles (green), MMP-9 (red), and SOX10 labeling schwannoma cells (magenta). Scale bars: top = 20 μm; middle = 50 μm; bottom = 100 μm. (B) Correlation between S8Z-AuNP fluorescence and tumor volume using Pearson correlation; **p = 0.01, *R* ^2^ = 0.99.

Measuring the activity of proteases is crucial, since they remain as inactive zymogens and require activation in specific tissue environments to function. However, traditional bulk zymography measures protease activity in homogenized tissue and lacks spatial resolution, and immunohistochemical assays measure protease abundance without distinguishing active enzymes from inactive zymogens. By contrast, *in situ* zymography directly visualizes protease activity within tissue sections while preserving the spatial context. By shifting from expression-based profiling to activity-based assay, our nanoparticle platform provides a more dynamic depiction of protease biology. Tyrosine kinase inhibitors (TKIs) like lapatinib and erlotinib,^48^ used in Phase I/II clinical trials for VS, rely on precise knowledge of tumor-specific tyrosine kinase receptor expression. In contrast, our platform offers a more adaptable approach by altering the peptide substrate to detect a broad range of proteases.^49^ This adaptability could enable the identification and monitoring of various proteolytic activities relevant to NF2. Furthermore, its modular architecture allows for the incorporation of stepwise-cleavable linkers or dual-enzyme activation mechanisms to refine the specificity and reduce off-target activation. Targeting motifs like pHLIP peptides, which bind activated macrophages in acidic microenvironments, can further improve specificity.^50–52^ While fluorescence detection supports early tumor identification, enzyme promiscuity limits specificity. Rationally engineered linkers, requiring sequential or concurrent cleavage by MMP-2 and MMP-9, can enhance specificity and restrict the prodrug activation to sites with coordinated protease activity. To improve nanoparticle distribution and retention, convection-enhanced delivery (CED) can be employed to provide pressure-driven infusion to enhance the local bioavailability.

### Visualization of MMP-9 Activity in Schwannoma *In Vivo*

Because MRI lacks sensitivity to detect biological changes during tumor progression, we developed an MMP-9-cleavable nanoparticle to enable real-time detection of tumor MMP-9 activity *in vivo*. We conjugated Cy5-labeled S8 or S23 as a control to AuNPs via 5 kDa PEG linkers, enabling MMP-9-mediated cleavage and fluorescence recovery of the Cy5-tagged peptide within tumor tissue (Figure 5A and Table S1). Tris-glycine gel analysis showed that free S8Q was degraded in serum, whereas S8Q-AuNP remained intact, indicating that AuNP conjugation protected the peptide from degradation (Figure S3). Following intratumoral injection in an NF2 schwannoma allograft model, S8Q-AuNPs showed 13.6-fold higher fluorescence than S23Q-AuNPs within 45 min (Figure 5B). This was further supported by a line trace analysis of fluorescence intensities of S8Q-AuNP and S23Q-AuNP, which revealed a significantly higher accumulation of S8Q-AuNPs (Figure 5C,D). ROC analysis demonstrated robust classification (AUC = 0.937; *p* = 0.043; Figure 5E). IF confirmed increased fluorescence with S8Q-AuNPs over S23Q-AuNPs (Figure 5F and Figure S4A). S8Q-AuNPs preferentially accumulated in the tumor with secondary uptake by the liver and spleen (Figure 5G). S8Q-AuNPs closely colocalized with MMP-9 and the cap-binding protein eIF4E^53^ in the tumor (Mander’s: 0.99 for MMP-9, 0.98 for eIF4E; Figure S4B,C). These results demonstrate that S8Q-AuNPs quantified tumor-associated MMP-9 activity *in vivo*.

**Figure 5:**
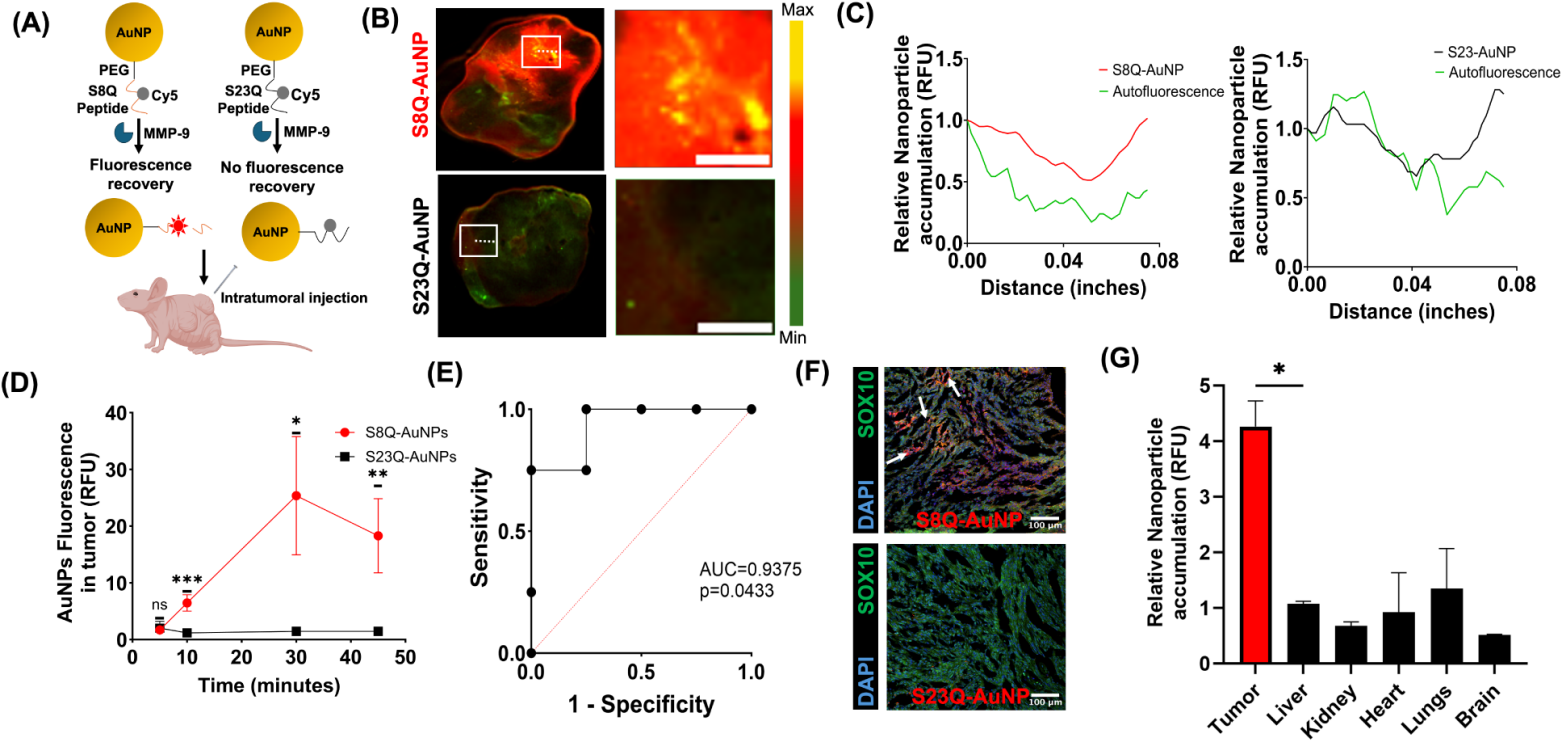
Visualization of MMP-9 activity in schwannoma *in vivo*. (A) Schematic illustration of S8Q-AuNP and S23Q-AuNP injected intratumorally into mice bearing schwannoma allografts (5 mg/kg); *n* = 6. (B) *Ex vivo* tumor fluorescence 45 min after injection. Scale bar = 0.05 in. (C) Line traces comparison: S8Q-AuNP (red, left panel), S23Q-AuNP (black, right panel), and autofluorescence (green, both panels). (D) Tumor fluorescence over 45 min. *p < 0.05; **p < 0.01; ***p < 0.001 by RM (Repeated Measures) ANOVA. (E) ROC analysis showing classification by S8Q-AuNPs (AUC = 0.93; *p* = 0.043). (F) IF staining of S8-AuNP and S23-AuNP accumulation in VS. Arrows indicate S8Q-AuNPs. DAPI (blue), SOX10 (green), nanoparticles (red). Scale bar: 100 μm. (G) Tissue and tumor biodistribution 45 min after S8Q-AuNP injection; *p < 0.05 by one-way ANOVA.

### Nanoparticle Sensing of MMP-9 Activity Predicts Schwannoma Growth

MMP-9 is increasingly recognized as a critical biomarker for tumor progression due to its role in tumor invasion and metastasis in various cancers.^33,34,54–56^ To evaluate the peptide-AuNPs as a diagnostic agent, *in vivo* fluorescence dynamics were assessed following systemic, intravenous administration in a mouse schwannoma allograft model, providing a clinically translatable framework to test the nanoparticle’s ability to detect MMP-9 activity (Figure 6A). Mice injected with S8Q-AuNPs exhibited significantly higher tumor fluorescence than those receiving S23Q-AuNPs (Figure 6B). After 4 h, fluorescence intensity of S8Q-AuNPs was substantially higher in the tumor (Figure 6C). To assess the correlation between the AuNP signal and tumor volume, mice were serially injected with nanoparticles on different days during tumor progression (Figure 6D). Between days 14 and 17, we observed a notable increase in the tumor before any appreciable change in tumor volume (Figure 6E,F). This early increase in MMP-9 activity suggests that nanoparticles could detect protease activity changes before apparent tumor growth. Fluorescence distribution of S8Q-AuNPs and S23Q-AuNPs was quantified at 24 h postinjection in different organs (Figure S5A,B). *Ex vivo* analyses of elemental gold by Inductively Coupled Plasma Mass Spectrometry confirmed nanoparticle accumulation correlated with fluorescence (Figure S5C).

**Figure 6:**
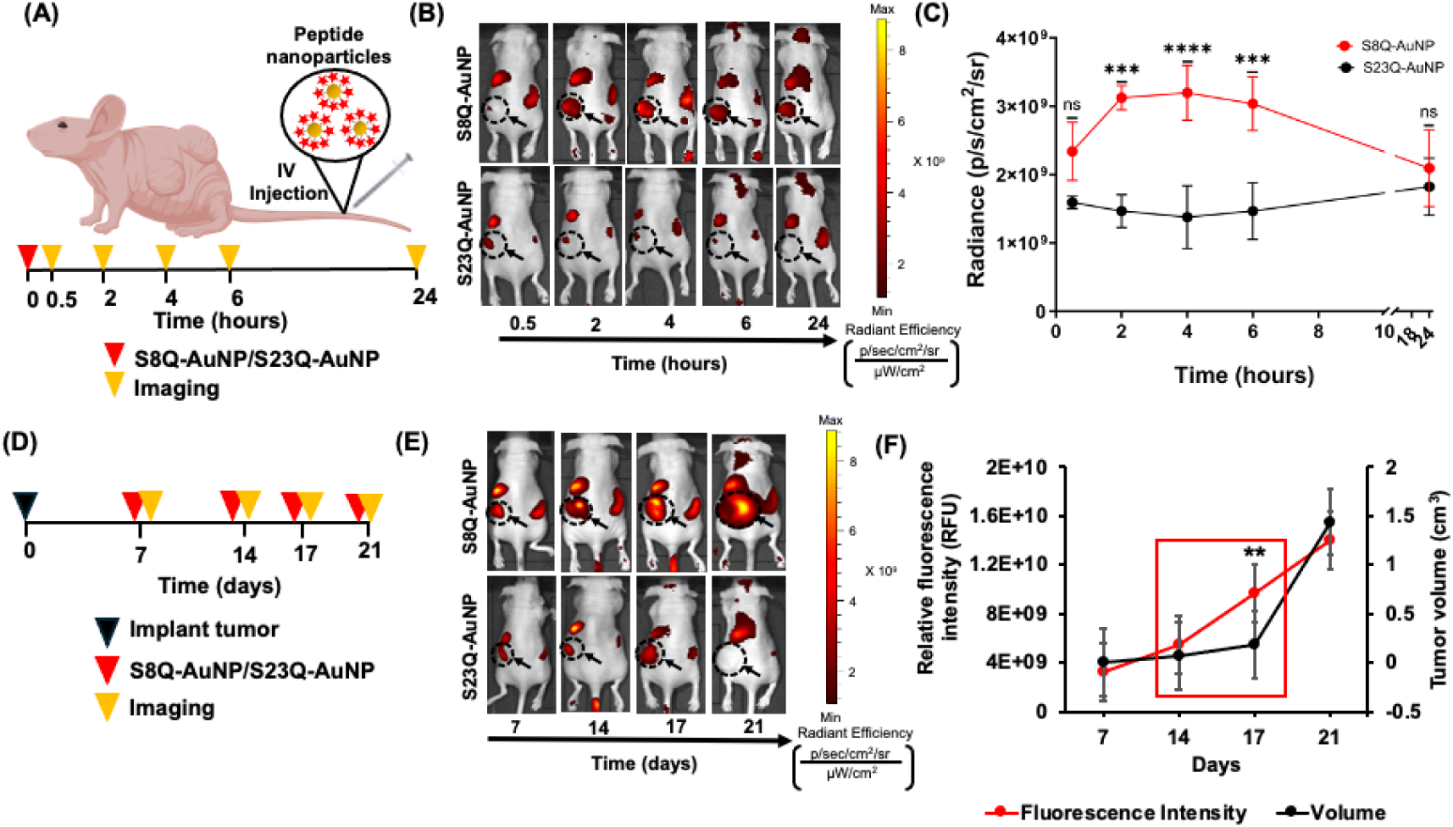
MMP-9 is a predictive marker of VS growth. (A) Schematic of the procedure. (B) *In vivo* whole-animal fluorescence of mice administered with S8Q-AuNPs and S23Q-AuNPs. (C) Quantification of epifluorescence radiant efficiency (*n* = 6) for S8Q-AuNP (red) and S23Q-AuNP (black) from 30 min to 24 h; ns, non-significant; ***p < 0.001; ****p < 0.0001 by one-way ANOVA. (D) Timeline for nanoparticle administration and imaging. (E) *In vivo* whole-animal fluorescence images of mice 4 h postinjection on different days (*n* = 6). (F) Quantitative analysis of fluorescence intensity of S8Q-AuNP (red) over tumor volumetric analysis (black). Red box indicates that between days 14 and 17, fluorescence change preceded a measurable increase in tumor volume. **p < 0.01 by one-way ANOVA.

MRI represents the gold standard for noninvasive monitoring of VS growth. However, its millimeter-scale resolution and the tumor’s slow progression can delay clinical detection of significant change by 1–2 years, resulting in missed treatment opportunities. Both conventional T2-weighted gadolinium-enhanced MRI and advanced techniques such as diffusion-weighted imaging (DWI) and dynamic contrast-enhanced (DCE) MRI offer only indirect insights into tumor growth via apparent diffusion coefficient (ADC) and microvascular imaging parameters,^57^ but they do not directly measure MMP-9 activity directly associated with tumor progression. In contrast, our nanoparticles can detect tumors as small as 2–3 mm, enabling earlier identification before clinically detectable growth, allowing for timely therapeutic interventions that target MMP-9 activity as a biomarker of tumor growth. To interpret this finding in the clinical context, we estimate VS to have a volumetric doubling time of 52.8 months from observed growth rates in retrospective patient cohorts.^58^ Assuming exponential growth, *N*_T_(t) = *N*_T,0_e­(ln2/[DT])^*t*^, where *N*_T_ represents the starting tumor size and DT the doubling time,^59^ we calculated mouse fluorescence-based and volume-based doubling times of 4.78 days and 5.53 days, respectively. Using these values, peptide-AuNPs could enable tumor detection 4.9 months earlier than MRI. By specifically targeting the accelerated growth phase of the tumor, typically between 7 and 14 mm, the nanosensor monitors tumor expansion via fluorescence doubling time, which, when extrapolated using assumed exponential kinetics to human VS progression, suggests that detection could occur up to 23 months earlier in patients. This is an approximate inference grounded in known human VS growth kinetics rather than a direct mouse-to-human extrapolation.^58^ This advancement may enable earlier interventions while patients still have good hearing. In summary, our results demonstrate that MMP-9 activity correlates with tumor growth, and AuNP-mediated MMP-9 sensing outperforms size-based measurements to monitor tumor progression. However, a detailed head-to-head comparison against established clinical standards such as MRI would require new animal cohorts, specialized imaging equipment, and a longitudinal study design.

### Association of MMP-9 with Invasion, Proliferation, and Angiogenesis in a Sciatic Schwannoma Model

Having verified that systemically administered AuNPs quantified MMP-9 activity in a subcutaneous schwannoma mouse model, we next assessed whether the same AuNPs could detect MMP-9 activity in a sciatic nerve schwannoma mouse model (Figure 7A). Following intravenous injection into mice bearing sciatic nerve schwannoma allografts, *in vivo* fluorescence imaging showed significantly higher Cy5 fluorescence in tumors treated with S8Q-AuNPs compared to that treated with S23Q-AuNPs (Figure 7B). Silver staining demonstrated enhanced nanoparticle accumulation at the tumor boundary (Figure 7C), with an intensified S8Q-AuNP fluorescence signal at the invasive front, which correlates with MMP-9 activity (Figure 7D), consistent with MMP-9’s known role in promoting tumor progression via ECM degradation. Tumor-associated macrophages (CD68+) and activated fibroblasts (α-SMA+) colocalized with S8-AuNPs compared to S23-AuNPs (Figure 7E and Figure S6). Cy5 signal from S8Q-AuNP also colocalized with markers of cell proliferation (Ki-67, 79.5% colocalization), angiogenesis (CD31, 93.1%), and apoptosis (cleaved caspase 3, 59.6%) (Figure 7F). Together, these data demonstrate that MMP-9 activity can be visualized with high spatial accuracy, identifying specific regions associated with tumor invasion, growth, angiogenesis, and apoptosis, demonstrating potential for early tumor detection.

**Figure 7:**
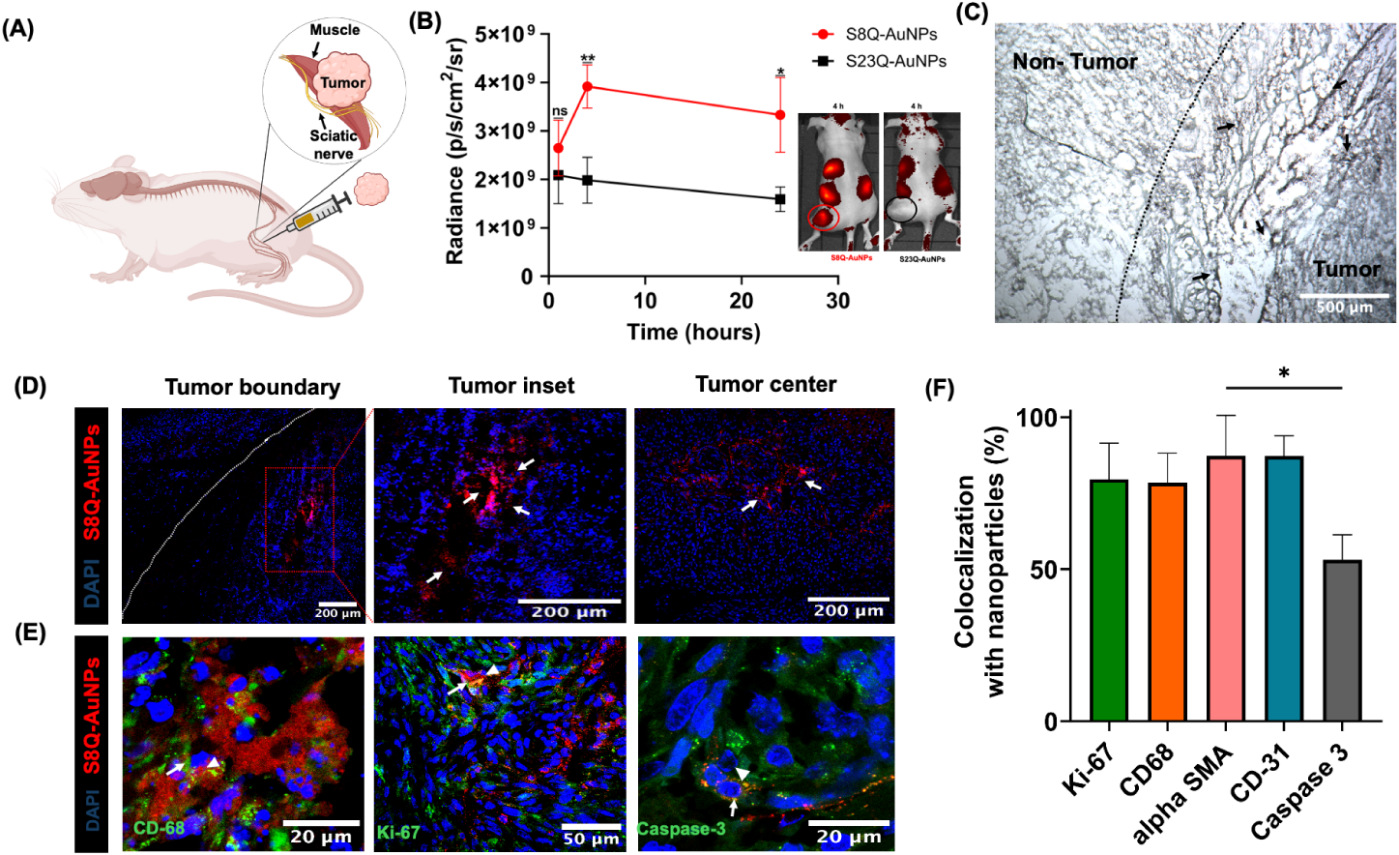
Detection of MMP-9 activity in a sciatic schwannoma model. (A) Schematic of the sciatic nerve schwannoma model. (B) Quantification of epifluorescence radiant efficiency and representative images *in vivo* 4 h after injection of S8Q-AuNPs (red) and S23Q-AuNPs (black) (5 mg/kg) into sciatic nerve tumor-bearing mice (*n* = 6). Statistical significance: ns = non-significant; *p < 0.05; **p < 0.01. (C) Silver staining revealed S8Q-AuNPs (arrows) accumulated at the margin between tumor and nontumor tissue. Scale bar = 500 μm. (D) IF of the tumor boundary, inset (red box), and center in frozen sections, with the tumor boundary indicated by a dotted line. Scale bar = 200 μm. DAPI (blue), S8Q-AuNP (red as indicated by arrows). (E) IF of tumors injected with S8Q-AuNPs (arrowheads) showing colocalization of S8Q-AuNP (red) with macrophages (CD68, green), proliferation marker (Ki-67, green), and apoptosis (cleaved caspase 3, green). (F) Quantification of the percentage of colocalization, *p < 0.05 by Kruskal–Wallis test followed by Dunn’s multiple comparison test.

Proteases play pivotal roles in both benign and malignant neoplasms by remodeling the ECM, promoting angiogenesis, and modulating immune cell infiltration that drives tumor proliferation and metastasis. MMP-9 activates pathways involving ECM remodeling and inflammatory signaling, such as TNF-α,^60^ while also regulating angiogenesis by mobilizing VEGF.^61^ Furthermore, MMP-9 degrades type IV collagen, creating migration pathways for tumor cells to amplify invasive capacity.^62^ MMP-9 also shapes the inflammatory microenvironment via increased neutrophil infiltration and contributes to immune evasion through the processing of CXCL11.^60^ Intratumoral regions with elevated MMP-9 correlate with invasive fronts in pancreatic and ovarian cancer.^54–56,63^ In NF2-associated VS, MMP-9 expression has been shown to correlate with tumor growth,^34,43^ and adherent VS demonstrated elevated MMP-9.^34^ Our data showed that MMP-9 activity is also elevated in nontumor cells such as macrophages, activated fibroblasts, and endothelial cells, where it potentially facilitates ECM remodeling and angiogenesis through VEGF mobilization, highlighting MMP-9 as a promising target for future therapies.

### Dynamic Monitoring of MMP-9 Activity during Drug Treatment

Because noninvasive, real-time monitoring of VS-associated MMP-9 remains limited, we evaluated the effectiveness of S8-AuNPs in tracking MMP-9 activity during therapy and correlating its inhibition with tumor growth suppression. Mice bearing subcutaneous schwannoma allografts were injected with MMP-9-IN-1 (20 mg/kg), while control mice received DMSO (Figure 8A). Tumor growth was markedly reduced in the MMP9-IN-I-treated group (Figure 8B). Fluorescence nanoparticle imaging revealed a marked reduction in the MMP-9 activity (Figure 8C). MMP-9 expression was confirmed to be reduced by 70% in MMP-9-IN-1-treated tumors (Figure 8D,E), with an increase in apoptosis (Figure 8F). These findings demonstrate that S8Q-AuNPs enable real-time imaging of MMP-9 activity in VS during protease inhibitor treatment and serve as an activity-based biomarker of therapeutic response.

**Figure 8:**
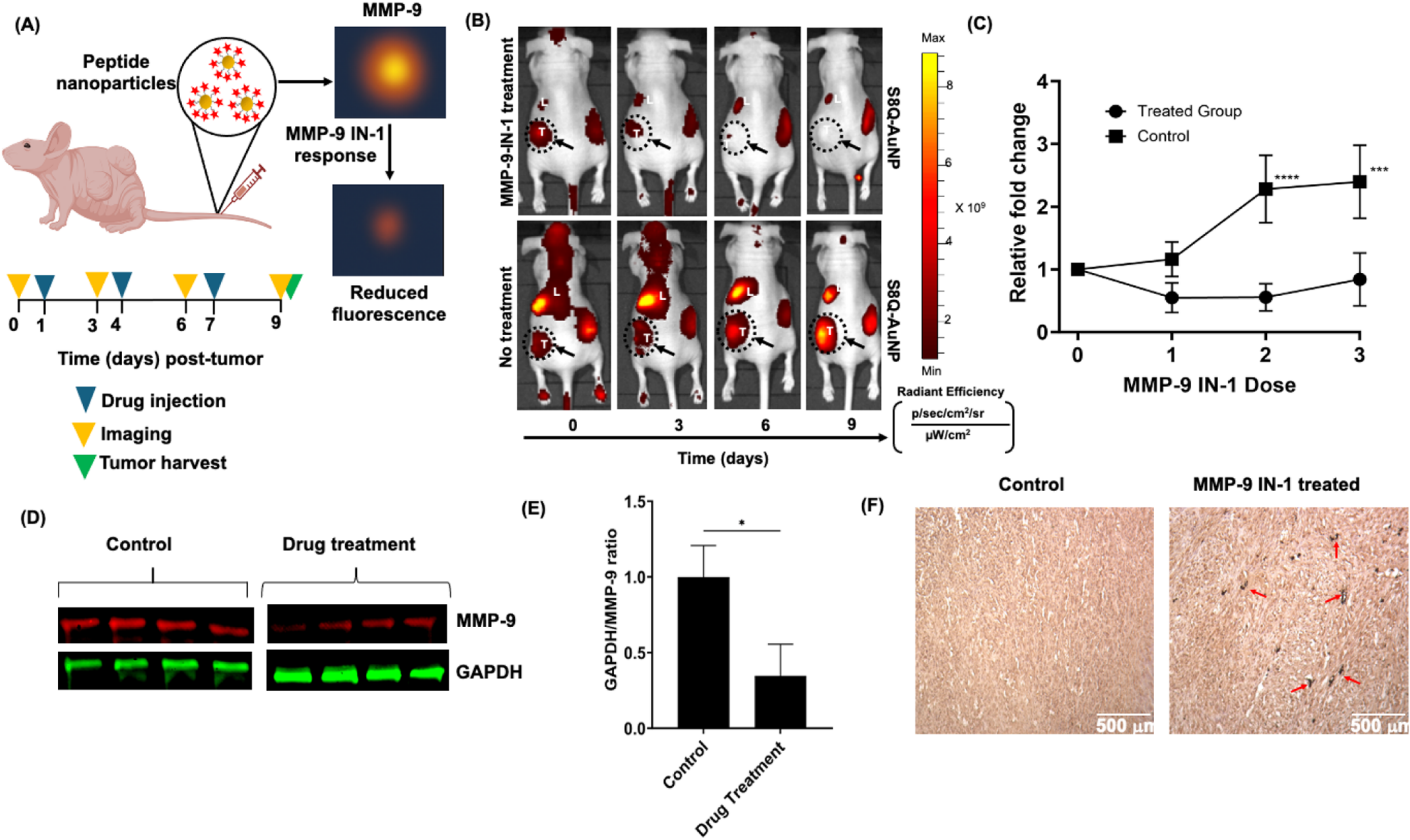
Therapeutic response monitoring in VS using S8Q-AuNPs. (A) Schematic of the study. S8Q-AuNPs are administered intravenously into mice bearing subcutaneous schwannoma allografts (*n* = 6). (B) *In vivo* fluorescence imaging of mice treated with MMP-9-IN-1 (20 mg/kg) or control (DMSO) over 9 days. MMP-9 activity was monitored using S8Q-AuNPs on days 0, 3, 6, and 9. (C) Quantification of relative S8Q-AuNP fluorescence intensity in the MMP-9-IN-1-treated group (circles) compared to control (squares), *n* = 6; ***p < 0.001; ****p < 0.0001 by one-way ANOVA. (D) Western blot analysis of MMP-9 expression in tumors (*n* = 4). (E) Quantification of MMP-9 protein expression normalized to GAPDH, *p < 0.05 by Mann–Whitney *U* test. (F) Immunohistochemical staining of cleaved caspase 3 (red arrows) from MMP-9-IN-1-treated mice and controls. Scale bar = 500 μm.

Bevacizumab, lapatinib, erlotinib, and brigatinib are commonly used drugs in NF2,^64–66^ with therapeutic responses typically monitored through MRI. However, MRI alone may not fully capture the biological effects of treatment. Plasma biomarkers such as free VEGF and placental growth factor correlate strongly with bevacizumab treatment response, reflecting vascular changes in NF2 patients.^67^ In sporadic, non-NF2 VS, biomarkers like S100B and MCP-3 show promise as indicators of tumor size and hearing preservation, respectively.^68^ These immune-related plasma markers provide valuable diagnostic and prognostic information. In contrast, our results suggest that this nanoparticle platform could be used for monitoring the response to MMP-9 inhibition *in vivo*. For instance, observing a reduction in MMP-9 activity could serve as an early indicator of the drug’s effectiveness, while assessing treatment impact on the microenvironment, such as reduced ECM remodeling, suppressed angiogenesis, and increased apoptosis, provides a more comprehensive assessment of therapeutic response. Additionally, persistently high MMP-9 levels could indicate treatment resistance and guide timely adjustments. Ultimately, this approach could optimize disease management by enabling more precise and responsive therapeutic decisions.

## Conclusion

Our study demonstrates that protease-responsive gold nanoparticles measure MMP-9 activity associated with VS, effectively differentiating between tumor and nontumor regions with remarkable sensitivity. The study further enhances our understanding of MMP-9’s role in VS progression. It highlights the central role of MMP-9 as both a biomarker and a therapeutic target through spatially resolved activity detection, presenting opportunities for earlier diagnosis and enhanced therapeutic monitoring. Integrating multiplexed peptide screening with cleavage-based *in situ* zymography assays offers a powerful framework for targeting protease dysregulation in NF2-associated neoplasms beyond VS.

## Methods

### Fluorescence Resonance Energy Transfer (FRET)-Based Screening of Protease Substrates

A fluorescence-based assay was used to screen a custom-designed peptide library for optimal MMP cleavage. The library was designed based on amino acid sequences containing protease-specific cleavage sites.^36,38,39,41,42,69–72^ Each peptide was produced by solid-phase peptide synthesis using standard FMOC (9-fluorenylmethoxycarbonyl) chemistry (CPC Scientific) and conjugated with appropriate fluorophore (5-FAM) and quencher (CPQ2) pairs. For the fluorescence cleavage assay, peptide substrates (3 μM) were incubated with recombinant proteases (10 nM) in a reaction volume of 100 μL in MMP buffer at 37 °C. Proteolytic cleavage of substrates was quantified by increase in fluorescence over time as measured by a fluorimeter (BioTek Cytation 1).

### Synthesis and Characterization of Peptide-Conjugated Gold Nanoparticles

AuNPs were synthesized using the citrate reduction method^73^ and functionalized with thiolated polyethylene glycol (PEG). Various lengths of PEG molecules were conjugated to AuNPs. The PEGylated AuNPs were then conjugated with protease-sensitive peptides at varying valencies using standard EDC-NHS chemistry.^74^ A peptide solution in 0.01 M sodium borate was added to PEG-AuNPs (1.5 nM in 0.01 M sodium borate buffer, pH 9). EDC (0.2 M) and sulfo-NHS (0.2 M) were added simultaneously for 24 h at room temperature. Dynamic Light Scattering (DLS) analysis was performed using a Zetasizer Nano ZS (Malvern), with a laser wavelength of 632.8 nm, measuring at a scattering angle of 173° and maintained at 25 °C. The concentration of AuNPs was determined by measuring absorbance at 520 nm and then using the Beer–Lambert Law.^75^

### Limit of Detection (LOD)

LOD was defined as the lowest enzyme concentration that yielded a fluorescence signal relative to the blank control. Recombinant MMP-9 and S8-AuNPs at varying concentrations ranging from 0 to 10 pg/mL were incubated in assay buffer at 37 °C for 30 min, and fluorescence intensity (520 nm) was recorded immediately thereafter.

### *In Vitro* Cellular Uptake Assays

MD-MSC cells were seeded in a 24-well plate until 70–80% confluency. A solution of 10 nM peptide-nanoparticles was prepared in fresh culture medium and added for 1–8 h. Cells were washed 3 times with PBS to remove uninternalized or nonspecifically bound nanoparticles. Cells were fixed with PFA, washed with PBS, and counterstained with DAPI. For MMP inhibition, Marimastat (1 mM, R&D Systems) was added 30 min prior to peptide-AuNPs incubation. Cells were stained with the following primary antibodies: monoclonal mouse anti-MMP-9 (Abcam, 1:200), monoclonal mouse Rab4 (BD Biosciences, 1:200), and monoclonal mouse Rab11 (BD Biosciences, 1:200) overnight at 4 °C, and corresponding fluorescently labeled secondary antibodies for 1 h at room temperature prior to mounting. The degree of colocalization between the peptide-AuNPs and endosomal markers was quantified using Mander’s coefficient (ImageJ, NIH).

For cytotoxicity, cells were seeded in a 96-well plate at a density of 10,000 cells per well until 70–80% confluency. Peptide-AuNPs (20 nM to 500 nM) in fresh culture medium were added for 24 h. Cells were washed three times with PBS. MTT reagent (5 mg/mL) was added for 4 h, and absorbance at 590 nm was measured (BioTek Cytation 1).

### *In Situ* Zymography

Freshly frozen tumor tissue sections from mouse schwannoma allografts and human vestibular schwannoma specimens were used for *in situ* zymography.^76^ Slides were fixed in ice-cold acetone for 10 min, air-dried, hydrated in PBS, and blocked in protease assay buffer (50 mM Tris, 150 mM NaCl, 5 mM CaCl_2_, 1 mM ZnCl_2_, pH 7.5) for 30 min at room temperature. Slides were incubated with AuNPs conjugated to either a specific protease-sensitive peptide (S8Z) or a noncleavable control peptide (dS8Z) for 4 h at 37 °C. For MMP inhibition, 1 mM of marimastat was added at the blocking and cleavage assay steps. For costaining experiments, the following primary antibodies were used: rabbit monoclonal SOX-10 (1:200, Novus), mouse monoclonal MMP-9 (1:200, Abcam), and rabbit monoclonal EIF4a (1:200, Novus). These were incubated overnight at 4 °C, followed by corresponding fluorescently labeled secondary antibodies, prior to confocal imaging (Olympus FV3000). For control, healthy mouse sciatic nerve tissue was obtained by euthanizing mice, followed by cervical dislocation to ensure pain-free collection. A midline incision was made to expose the sciatic nerve, which was carefully harvested to avoid damage. This harvested nerve was then used as control.

### Measurement of Peptide-AuNps Fluorescence and Tumor Growth

After tumor establishment, animals received intravenous injections of S8Q AuNP or S23Q AuNP (5 mg/kg). Mice were imaged at different time points after peptide-AuNP injection and at days 3, 7, 14, 17, and 21. Optical images were acquired with IVIS Spectrum (PerkinElmer, Waltham, MA, USA) in fluorescent modality with an excitation filter of 710 nm and an emission filter of 800 nm. Measurements were done by tracing a region of interest (ROI) on the fluorescent images in correspondence with the tumor region of the animals. After the last day of acquisition, mice were sacrificed, and the liver, kidney, spleen, lungs, heart, and brain were excised and imaged (LI-COR, Lincoln, NE, USA). The fluorescence intensity of Cy5 was normalized against autofluorescence at 800 nm by using ImageJ.

## Supplementary Material



## Notes

Additional methods; peptide sequences; data including characterization of peptide-AuNPs, confocal microscopy images, peptide degradation assay, nanoparticle distribution, and ICP-MS analysis; statistical analysis (PDF)
