# Supplementary material for: Matrix Metalloproteinase‑9 (MMP-9) Activatable Gold Nanoparticles for In Situ Zymography and Diagnostics of Neurofibromatosis Type 2 (NF2) Tumors
Source: ACS Appl Nano Mater. 2026 Jan 23;9(5):2245–57. doi: 10.1021/acsanm.5c04657 (PMC12887933; doi:10.1021/acsanm.5c04657)
Supplement: Supplementary file 1 [file an5c04657_si_001.pdf]

Supporting information

Matrix Metalloproteinase-9 (MMP-9) Activatable Gold Nanoparticles for In situ Zymography and Diagnostics of Neurofibromatosis Type 2 (NF2) Tumors

Shimayali Kaushal[1], PhD; ThiNgocHan Nguyen[1], PhD; Melanie Fisher[1], BS; Hsuan-Chih Kuo[1], BS; Zachary D. Schultz[2], PhD; Yin Ren[1*], MD PhD

[1]Department of Otolaryngology–Head and Neck Surgery, Division of Otology, Neurotology and Cranial Base Surgery, The Ohio State University Wexner Medical Center, Columbus, Ohio, USA

[2]Department of Chemistry and Biochemistry, The Ohio State University, Columbus, Ohio, USA

*Corresponding Author: Yin Ren, MD, PhD
Department of Otolaryngology–Head and Neck Surgery
The Ohio State University
915 Olentangy River Rd Ste 4000
Columbus, OH 43212 USA
Email: ren.yin@gmail.com

Patient characteristics

All samples used in the study had appropriate institutional review board (IRB) approval from the Ohio State University Wexner Medical Center (IRB#1994H0241). All patients were informed of the research study on their pre-operative clinic visit, typically 1-2 months prior to surgery. On the day of surgery, informed consent was obtained from all study participants. Inclusion criteria included age older than 18 years, had a diagnosis of vestibular schwannoma, no history of radiation and had complete medical records. Patients with a diagnosis of nonvestibular schwannoma or meningioma, treated with radiation prior to surgery, or incomplete records were excluded. Tumor volume was calculated using the modified ellipsoid formula: $4/3\pi \times ½(W \times L \times H)$ where W, L and H represent the width, length and height of the tumor in all three axes from the most recent, contrast-enhanced, T1-weighted MRI axial and coronal slices prior to surgery. For tumors that extended into the cerebellopontine angle, size measurements included only the cisternal portion.

Sample collection and processing

Tumors from surgical debulking are transported from the operating suite to the pathology lab and immediately placed in fresh cold DMEM media where it was cataloged and weighed. Tumors are minimally manipulated to maintain tissue architecture. An independent neuropathologist verified the diagnosis and presence of sufficient vestibular schwannoma tissue on each section. A portion of the specimen was flash frozen in optimum cutting temperature (OCT) solution and stored at -80°C until further use. Histological sections 7 μm in thickness were cryosectioned for in situ zymography and immunofluorescence studies.

Cell line

A mouse neurofibromatosis type 2 (NF2)-deficient (NF2-/-) schwannoma cells (MD-MSC) were used for developing tumors in mice models [gift from Dr. L. Xu, Massachusetts General Hospital] [1]. The cell lines were infected with lentivirus encoding secreted Gaussia luciferase (Gluc). MD-MSCs were maintained in Dulbecco's Modified Eagle's Medium (DMEM) supplemented with 10% fetal bovine serum (FBS) and 1% penicillin/streptomycin. Cells were maintained in a humidified incubator at 37°C with 5% CO_2.

Transmission Electron Microscopy (TEM)

Peptide-AuNPs were suspended in deionized water, placed on a copper grid coated with a thin carbon film and allowed to dry. The TEM microscope (Tecnai 20) was operated at 80kV from 50,000x to 200,000x.

Gel Retardation assay

A 2% agarose gel was prepared using Tris-acetate-EDTA buffer and loaded with peptide-AuNPs and PEG-AuNPs. The retardation of the peptide-AuNPs in comparison to the PEG-AuNPs was analyzed by measuring the distance traveled by each formulation. Each formulation was prepared in triplicate (n = 3 samples), for consistency.

Mouse schwannoma allograft model

Athymic nude mice aged 6 to 8 weeks were sourced (OSU TVAR facility, not Jax Labs) in accordance with the protocol approved by The Ohio State University Institutional Animal Care and Use Committee (IACUC) (2022A00000043). All animal studies were performed with approval from IACUC (#2022A00000043) and in compliance with institutional guidelines. MD-MSC cells (5000 cells in 10 μL) Matrigel (BD Biosciences) were injected subcutaneously into the flank. Tumor volume was assessed every other day using the ellipsoid formula $4/3\pi\times½(W\times L\times H)$ where W, L and H represent the width, length and height of the tumor in all three axes. In the sciatic nerve allograft model, athymic nude mice aged 6 to 8 weeks were anesthetized and the surgical field was prepared as described [2]. Following skin incision, the sciatic nerve was carefully dissected and exposed, and up to 10,000 cells in Matrigel was

injected into the sciatic nerve sheath using a 27G Hamilton syringe. Tumor growth was regularly monitored for up to two weeks by bioluminescence. Briefly 10 µl of whole blood was collected from tail vein and mixed with 10 µl of EDTA immediately to avoid clotting. Blood sample was transferred to a 96-well plate, mixed with coelenterazine substrate and Gluc activity was measured using a bioluminescence (Biotek, Cytation1) plate reader.

For evaluation of therapeutic effects of MMP-9 inhibition, MD-MSC cells (5000 cells in 10 µL) Matrigel were injected subcutaneously into the flank of 6 to 8 weeks old female nude mice. Mice were randomized into two different groups comprising 4 animals each. The groups were: control group receiving saline and treatment group receiving MMP-9-IN-1 at 20 mg/kg. Animals were injected with nanoparticles after day 7 of tumor cell implantation. Mice were weighed and tumor volumes were measured every other day and calculated by the ellipsoid formula [4/3 pi × L/2 × W/2 × H/2].

Inductively Coupled Plasma Mass Spectrometry (ICP-MS)

Inductively Coupled Plasma Mass Spectrometry (ICP-MS) was utilized to investigate the gold biodistribution of peptide-AuNPs. Organs and tumors were weighed, digested using nitric acid (70% v/v) and ICP-MS was performed by introducing samples into an argon plasma, where the AuNPs were ionized and analyzed based on their mass-to-charge ratios. The concentration of gold was quantified and normalized by weight.

Statistical Analysis

In this study, statistical analyses were performed to assess differences between groups using a two-tailed Student's T-test for normally distributed data and a Mann-Whitney U test for non-normal distributions in binary comparisons. A non-parametric one-way ANOVA followed by Dunnett's multiple comparisons test and non-parametric two-way ANOVA with Tukey's multiple comparisons test were employed. The diagnostic accuracy of our nanoprobe was assessed through Receiver Operating Characteristic (ROC) analysis. All statistical analysis of immunofluorescence staining was performed using GraphPad Prism

and a significance threshold of $p < 0.05$ was considered statistically significant. A power analysis was conducted using an effect size of 0.5, variability of 1.0, a significance level (α=0.05), and a desired power of 80%, indicating a total of 6 animals per group would be required to ensure adequate power to detect significant effects.

References

(1) Wu, L.; Vasilijic, S.; Sun, Y.; Chen, J.; Landegger, L. D.; Zhang, Y.; Zhou, W.; Ren, J.; Early, S.; Yin, Z.; et al. Losartan prevents tumor-induced hearing loss and augments radiation efficacy in NF2 schwannoma rodent models. *Sci Transl Med* **2021**, *13* (602). DOI: 10.1126/scitranslmed.abd4816 From NLM Medline.

(2) Lee, J. K.; Sobel, R. A.; Chiocca, E. A.; Kim, T. S.; Martuza, R. L. Growth of human acoustic neuromas, neurofibromas and schwannomas in the subrenal capsule and sciatic nerve of the nude mouse. *J Neurooncol* **1992**, *14* (2), 101-112. DOI: 10.1007/BF00177613 From NLM Medline.

(3) Soleimany, A. P.; Kirkpatrick, J. D.; Su, S.; Dudani, J. S.; Zhong, Q.; Bekdemir, A.; Bhatia, S. N. Activatable Zymography Probes Enable In Situ Localization of Protease Dysregulation in Cancer. *Cancer Res* **2021**, *81* (1), 213-224. DOI: 10.1158/0008-5472.CAN-20-2410 From NLM Medline.

Supplementary Table S1. Peptide sequences used in the study.

Substrate	Sequence	Readout
S1	Mca-Pro-Leu-Gly-Leu-Dpa-Ala-Arg-NH2. AcOH	Fluorescence (in vitro)
S3	Mca-Pro-Leu-Ala-Nva-Dap(Dnp)-Ala-Arg-NH2	Fluorescence (in vitro)
S4	Mca-Arg-Pro-Pro-Gly-Phe-Ser-Ala-Phe-Lys(Dnp)-OH	Fluorescence (in vitro)
S5	Dnp-P(Cha)G(CMe)HA(KNma)-amide	Fluorescence (in vitro)
S6	Mca-Pro-Leu-Ala-Cys(p-OMeBz)-Trp-Ala-Arg(Dpa)-NH_2	Fluorescence (in vitro)
S8	CPQ2-Gly-Pro-Leu-Ala-Tyr-Trp-Ala-Arg-Lys(5FAM)-PEG2-DLys-Cys-NH2	Fluorescence (in vitro)
S10	CPQ2-Gly-Pro-Leu-Gly-Val-Arg-Gly-Lys(5FAM)-PEG2-DLys-Cys-NH2	Fluorescence (in vitro)
S11	CPQ2-Gly-Pro-Gln-Gly-Ile-Trp-Gly-Gln-Lys(5FAM)-PEG2-DLys-DLys-Cys-NH2	Fluorescence (in vitro)
S12	CPQ2-Pro-Leu-Gly-Leu-DPhe-Ala-Arg-Lys(5FAM)-PEG2-Gly-Cys-NH2	Fluorescence (in vitro)
S13	CPQ2-Arg-Ala-Met-His-Met-Tyr-Lys(5FAM)-PEG2-DLys-Cys-NH2	Fluorescence (in vitro)
S14	CPQ2-Arg-Ser-Glu-Asn-Ile-Arg-Lys(5FAM)-PEG2-Gly-Cys-NH2	Fluorescence (in vitro)
S15	CPQ2-Arg-Ile-Gly-Phe-Leu-Arg-Lys(5FAM)-PEG2-Gly-Cys-NH2	Fluorescence (in vitro)
S16	CPQ2-Ala-Lys-Val-Arg-Gly-Lys(5FAM)-PEG2-Gly-Cys-NH2	Fluorescence (in vitro)
S17	CPQ2-Ala-Phe-Gly-Arg-Gly-Lys(5FAM)-PEG2-Gly-Cys-NH2	Fluorescence (in vitro)
S18	CPQ2-Arg-Ser-Cit-Gly-Homophe-Tyr-Leu-Tyr-Lys(5FAM)-PEG2-DLys-Cys-NH2	Fluorescence (in vitro)
S19	CPQ2-Gln-Glu-Val-Tyr-Gly-Ala-Met-Pro-Lys(5FAM)-PEG2-DLys-DLys-Cys-NH2	Fluorescence (in vitro)
S20	CPQ2-DPhe-Pro-Arg-Ser-Gly-Gly-Gly-Lys(5FAM)-PEG2-DLys-Cys-NH2	Fluorescence (in vitro)
S21	CPQ2-Gly-Tyr-Val-Ala-Asp-Ala-Pro-Lys(5FAM)-PEG2-DLys-DLys-Cys-NH2	Fluorescence (in vitro)
S22	CPQ2-Gly-Pro-Leu-Gly-Leu-Arg-Ser-Trp-Lys(5FAM)-PEG2-DLys-Cys-NH2	Fluorescence (in vitro)
S23	CPQ2-Gly-Leu-Val-Pro-Arg-Gly-Ser-Gly-Lys(5FAM)-PEG2-DLys-Cys-NH2	Fluorescence (in vitro)
S24	CPQ2-Gly-Gly-Ser-Gly-Arg-Ser-Ala-Asn-Ala-Lys-Gly-Lys(5FAM)-PEG2-Gly-Cys-NH2	Fluorescence (in vitro)
S25	CPQ2-Gly-Ile-Leu-Ser-Arg-Ile-Val-Gly-Gly-Gly-Lys(5FAM)-PEG2-DLys-Cys-NH2	Fluorescence (in vitro)
S26	CPQ2-Gly-Asp-Gln-Gly-Ile-Ala-Gly-Phe-Lys(5FAM)-PEG2-DLys-DLys-Cys-NH2	Fluorescence (in vitro)
S8-Z	U-eeeeeeeee-X-GPLAYWAR-rrrrrrrrr-X-K(5FAM)-NH2	Fluorescence (in situ)

dS8-Z	U-eeeeeeeee-X-Gplaywar-rrrrrrrrr-X-K(5FAM)-NH2	Fluorescence (in situ)
S8-Q	(QSY21)-GPLAYWAR-K(Cy5)-PEG2-Dlys-C-NH2	Fluorescence (in vivo)
S23-Q	(QSY21)-GLVPRGSG-K(Cy5)-PEG2-Dlys-C-NH2	Fluorescence (in vivo)

Abbreviations:

NH2: amidated C-terminus

PEG2: diethylene-glycol

Lowercase sequence: d-stereoisomer

5FAM-CPQ2: FRET pair, with 5-Carboxyfluorescein as fluorophore and CPQ2 as quencher

QSY21-Cy5: FRET pair, with Cyanin5 as fluorophore and QSY21 as quencher

Q= quenched, Z= zymography

U= succinoyl

X= 6-aminohexanoyl

Supplementary Table S2:

MMP-9 Concentration (pg/mL)	Mean Fluorescence (a.u.)	Standard Deviation	RSD (%)
0	0	0	0
1	0.10366667	0.00188562	1.818924196
10	5.087	0.0008165	0.01605065
20	10.0836667	0.0004714	0.004674932
50	29.084	0.00163299	0.005614747
100	70.086	0.00141421	0.002017826

Relative standard deviation (RSD). Values calculated from three independent replicates for each MMP-9 concentration. Values <5% indicates excellent precision.

Figures

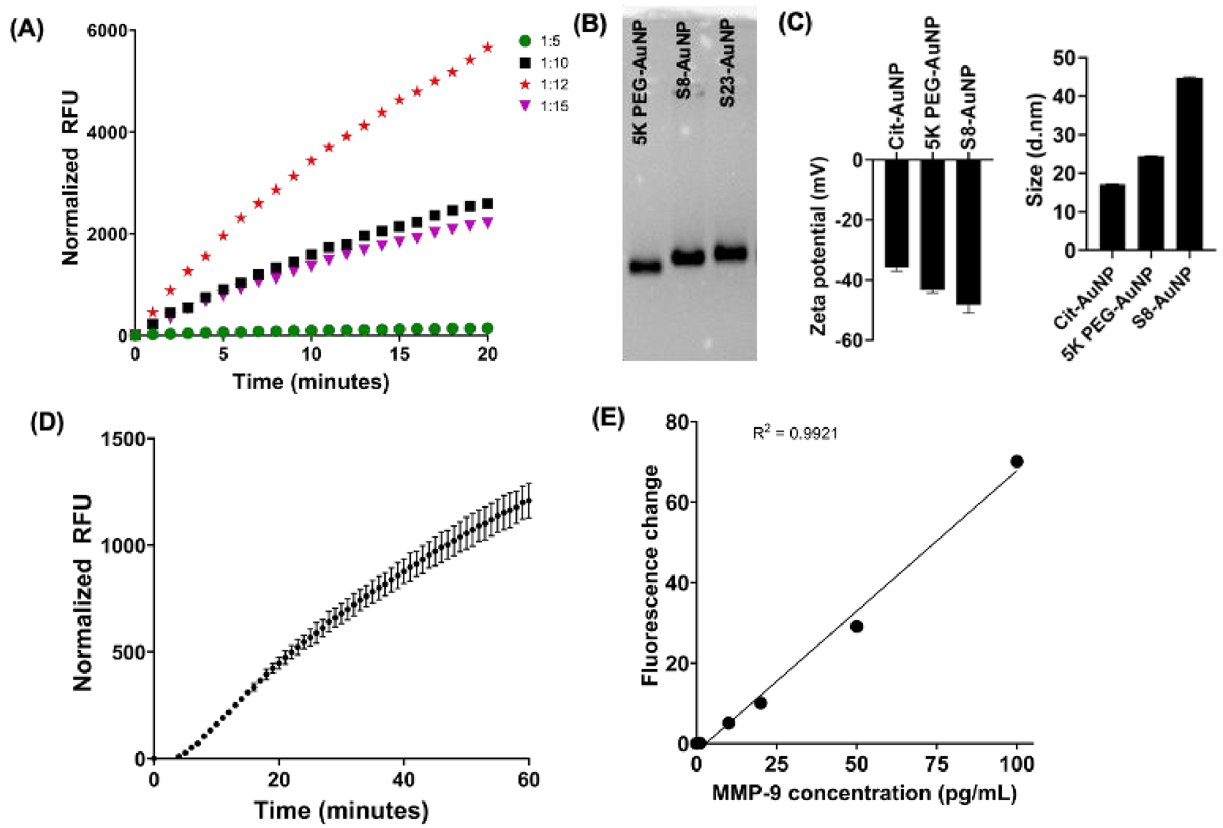

Fig S1. (A) Effect of peptide valency on MMP-9 induced fluorescence of S8-AuNPs. (B) Gel retardation assay showing peptide conjugation with AuNPs. (C) Zeta potential and size of different AuNPs before and after each conjugation. (D) Time dependent fluorescence activation curve of S8-AuNP in the presence of MMP-9. (E) Linear fluorescence response of the S8-AuNP sensor toward MMP-9.

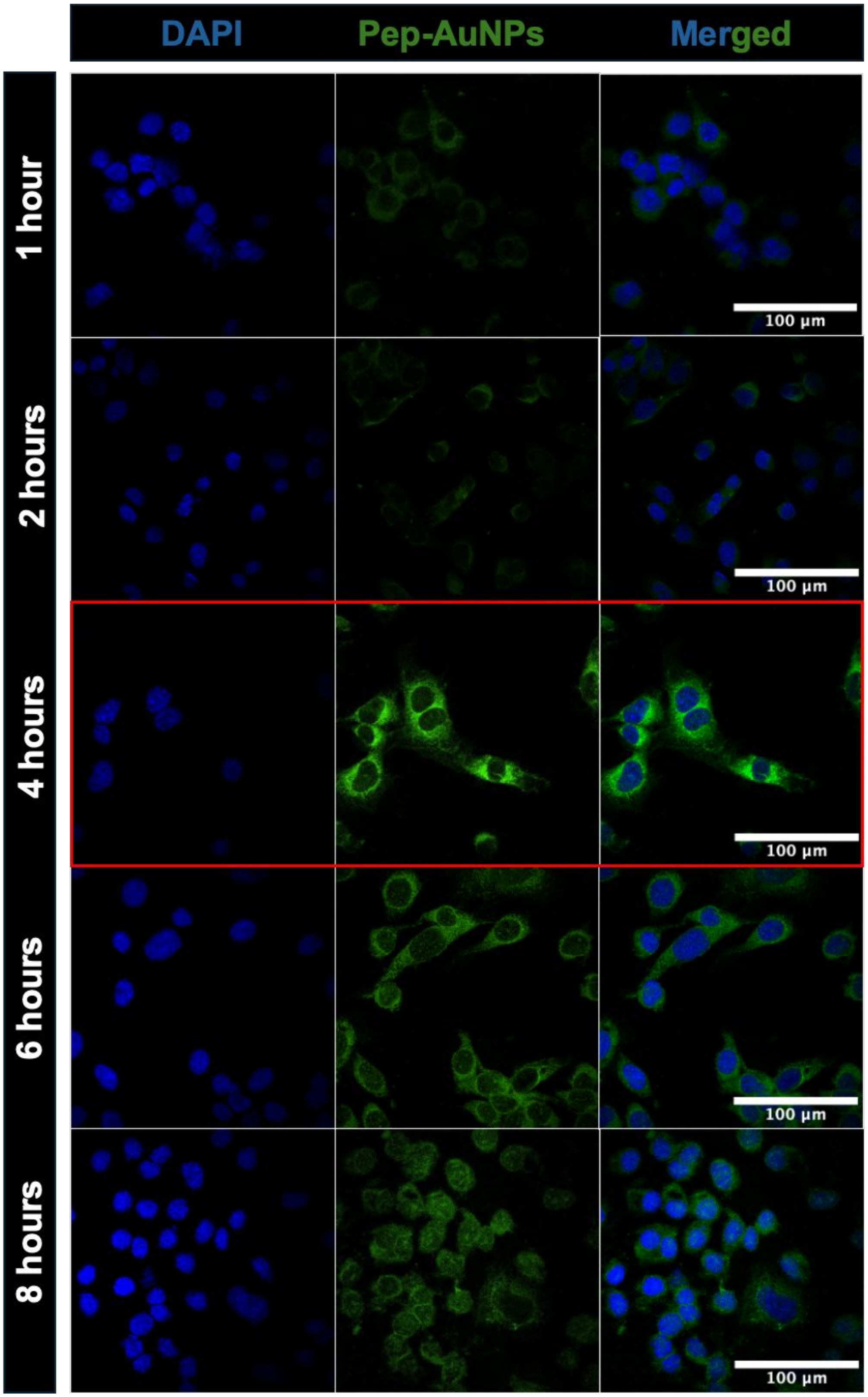

Fig S2. IF images of cellular uptake of S8-AuNPs in MD-MSC cell line at different time points. DAPI, blue; nanoparticle, green. All scale bars, 100 µm.

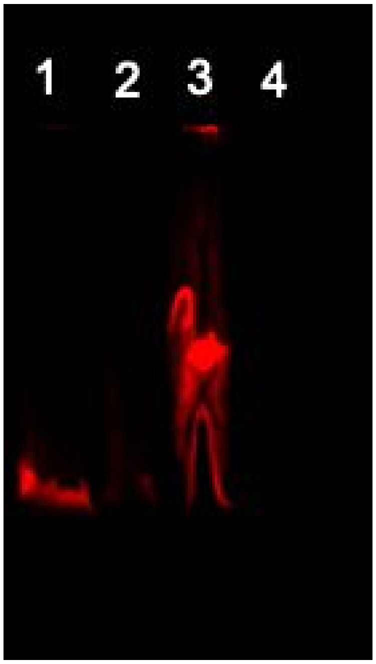

1: Peptide-AuNPs (without PEG)

2: Peptide-PEG-AUNPs

3: Peptide alone

4: Serum alone

Fig S3. Peptide degradation assay demonstrating that PEG-AuNP-conjugated peptide exhibits the highest stability compared to peptide-AuNPs without PEG and free peptide.

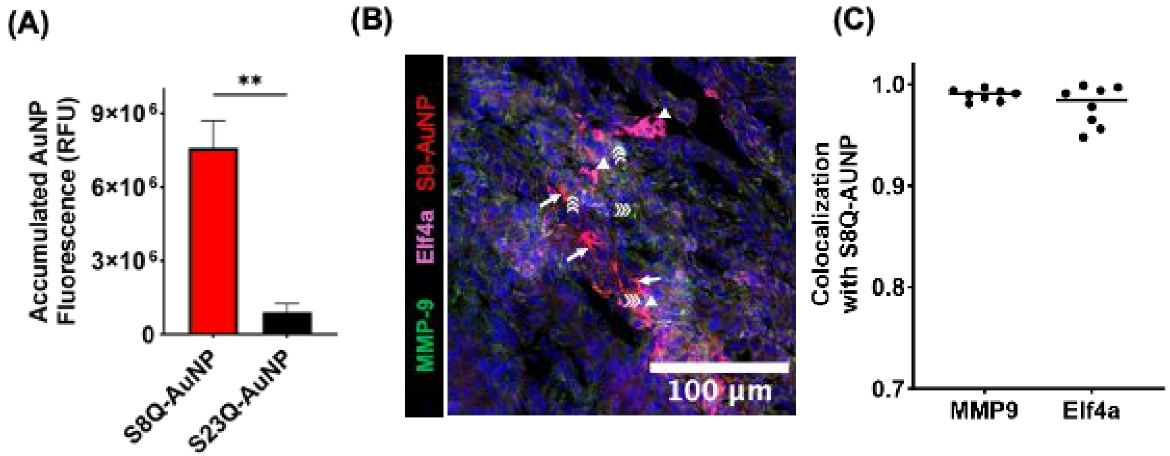

Fig S4. (A) Bar graph illustrates differences in tumor nanoparticle fluorescence over time. (B) IF imaging reveals S8Q-AuNP (red, arrows) co-localized with MMP-9 (green, arrowheads) and Eif4a (pink, triple arrowheads). Scale bar, 100 um. (C) Mander's analysis of S8Q-AuNP colocalization with MMP-9 and Eif4a.

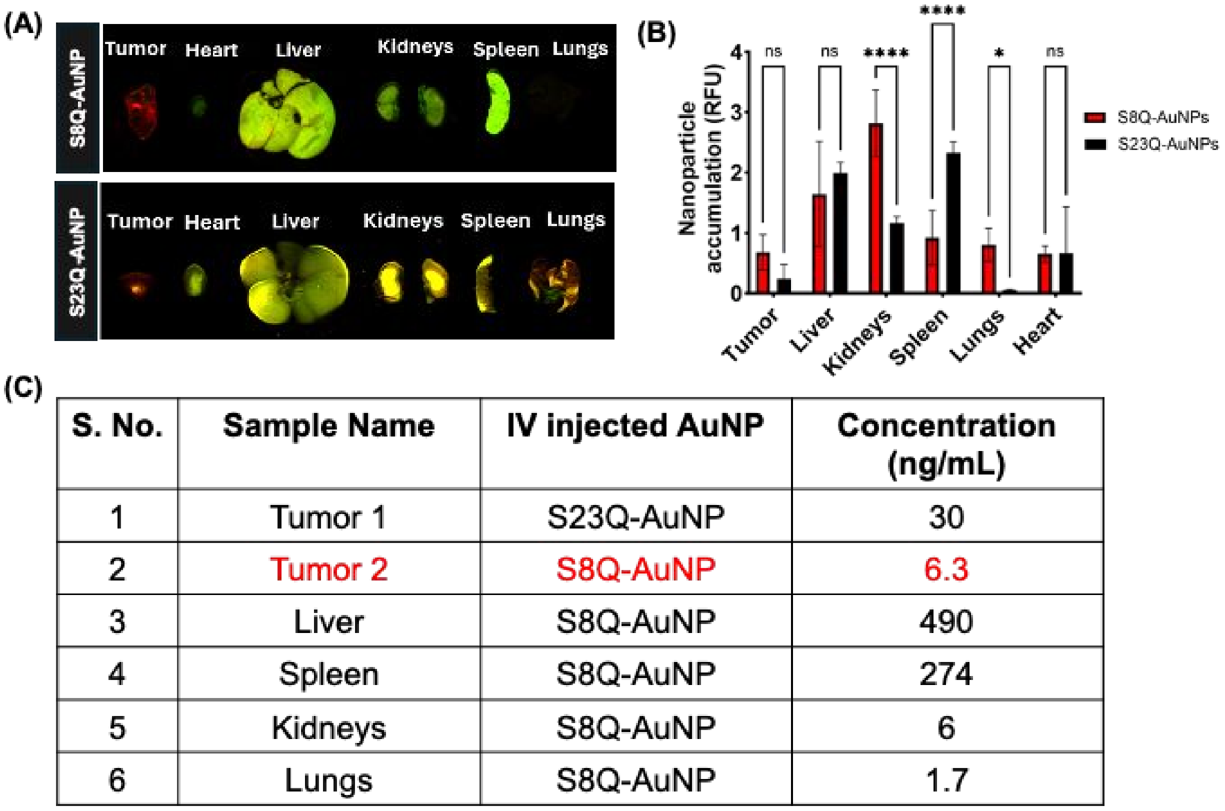

(C)

S. No.	Sample Name	IV injected AuNP	Concentration (ng/mL)
1	Tumor 1	S23Q-AuNP	30
2	Tumor 2	S8Q-AuNP	6.3
3	Liver	S8Q-AuNP	490
4	Spleen	S8Q-AuNP	274
5	Kidneys	S8Q-AuNP	6
6	Lungs	S8Q-AuNP	1.7

Fig S5. (A) Fluorescence distribution across various organs 24 hours post-injection. Red indicates nanoparticle accumulation. (B) Quantitative comparison of nanoparticle accumulation in different organs between S8Q- and S23Q-conjugated AuNPs. (C) ICP-MS analysis of tumor and organs.

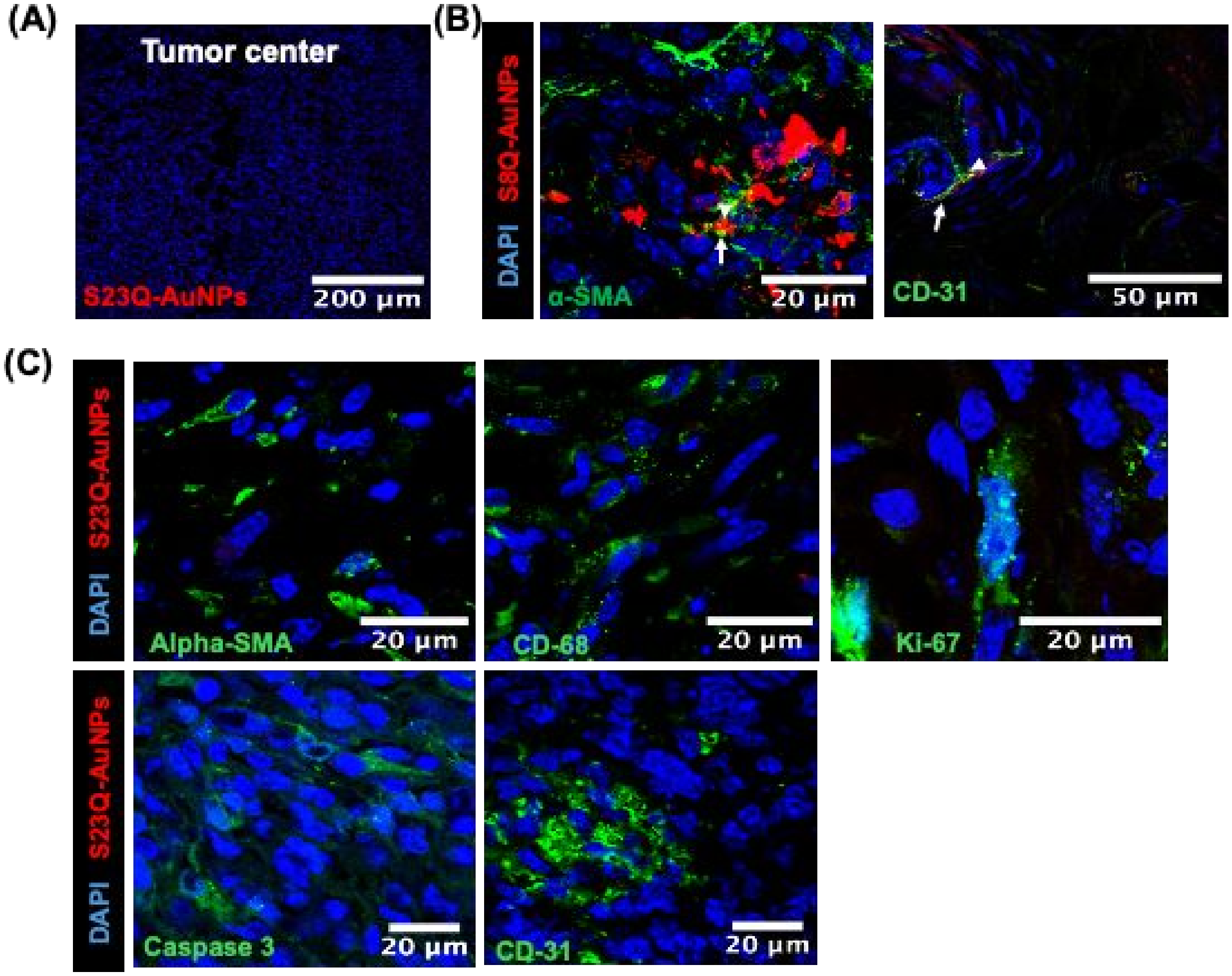

Fig S6. (A) IF imaging of the tumor center in fresh frozen sections; sections were stained for DAPI (blue), scale bar=100 µm, and showed no S23Q-AuNP fluorescence. (B) Co-staining of S8Q-AuNPs with fibroblasts (alpha SMA) and angiogenesis (CD-31). (C) IF staining of mouse sciatic tumor tissues showing markers of fibroblasts (α-SMA), macrophages (CD68), proliferation (Ki-67), angiogenesis (CD31), and apoptosis (cleaved caspase-3). No co-localization was observed with S23Q-AuNPs (red).
